# Supplementary material for: PREHAB FAI- Prehabilitation for patients undergoing arthroscopic hip surgery for Femoroacetabular Impingement Syndrome -Protocol for an assessor blinded randomised controlled feasibility study
Source: PLoS One. 2024 Apr 11;19(4):e0301194. doi: 10.1371/journal.pone.0301194 (PMC11008823; doi:10.1371/journal.pone.0301194)
Supplement: S1 Appendix — (DOCX) [file pone.0301194.s001.docx]

**S1 Appendix. Details of the prehabilitation intervention**

The prehabilitation programme is based on the International consensus statement as explained earlier and its delivery described using TIDieR checklist. The six key components targeted will include;

1. Muscle strength
2. Range of motion
3. Proprioception
4. Cardiovascular fitness
5. Address co-existing pathologies (e.g., gluteal, adductor tendinopathy) if any
6. Patient education to alleviate anxiety and better prepare for surgery

**Prehabilitation Phase 1 (0-4 weeks)**

This phase consists of 1 educational session (45 min) in a group setting, 2 fortnightly one to one session with a qualified Physiotherapist and 1 remotely supervised session weekly using Telehealth. Additionally, participants will be asked to carry on with an unsupervised home exercise programme twice weekly. Adherence will be monitored using Physitrack app.

**Prehabilitation Phase 2 (5-8 weeks)**

This phase consists of 2 fortnightly one to one session with a qualified Physiotherapist and 1 remotely supervised session weekly using Telehealth. Additionally, participants will be asked to carry on with an unsupervised home exercise programme twice weekly. Adherence will be monitored using Physitrack app.

**Prehabilitation for FAI surgery- Phase 1 (0-4 weeks)**


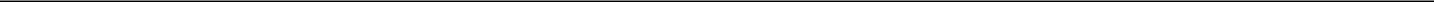


2 Sets / 1 Rep / 10 min duration / 100 bpm / 3 RPE

**1. Stationary cycle spin bike- Cardiovascular fitness**


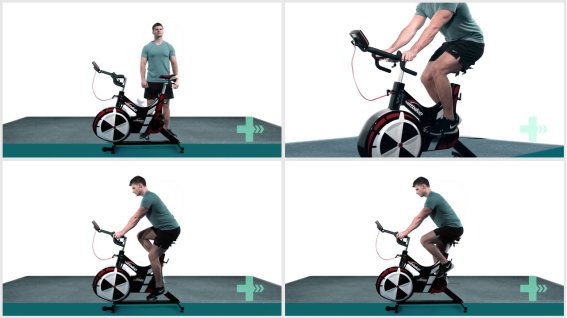


Adjust the bike seat and settings as required and commence cycling.

*Aim for a minimum of 150 minutes of moderate CV exercises per week-Swimming, Cycling, Flat terrain walking, Elliptical cross trainer*


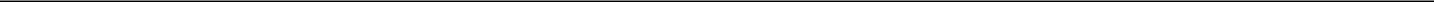


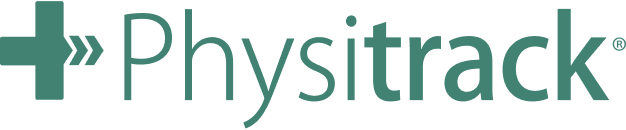


2 Sets / 10 Reps / 5 s hold

**2. Bridging – simple- All Gluteals**


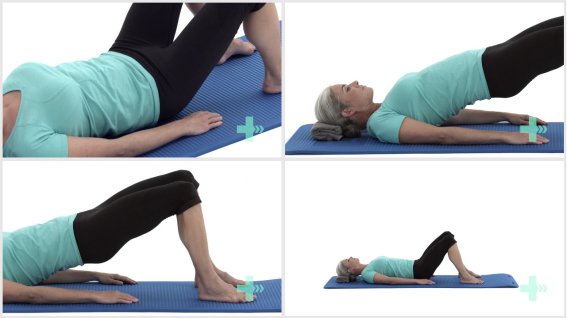


Lie on your back with your knees bent and your feet flat on the floor.

Gently tilt your pelvis as if you are imprinting your lower back into the floor and lift your hips up into the air while still holding your pelvis level.

Hold in the bridge position before you then lower, keeping your navel drawn in and slowly lowering your spine back down onto the floor, one vertebrae at a time. Keep your buttocks tight, until your pelvis rests back down on the floor.


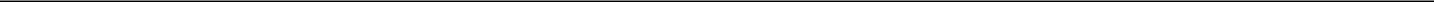


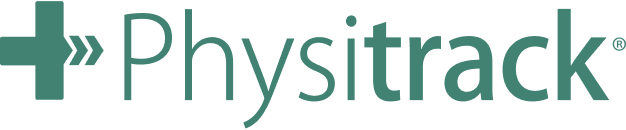


2 Sets / 10 Reps

**3. Bridge with resisted hip abduction- Hip abductors**


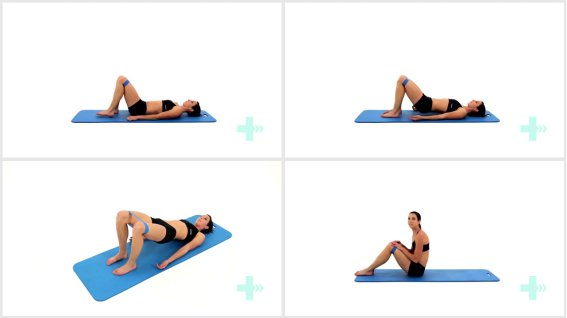


Place a resistance band loop around both thighs, just above your knees.

Lie on your back with your knees bent and feet flat on the floor. Place legs hips width apart so that there is tension in the band. Raise your hips up into a bridge, keeping the knees hips width apart. Control the movement back down to the start position, maintaining constant tension on the band.


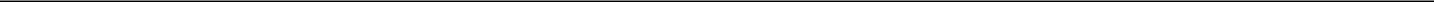


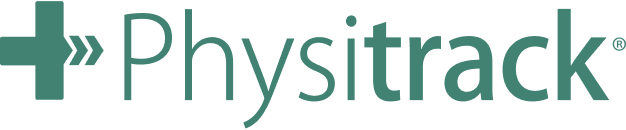


2 Sets / 10 Reps / 5 s hold

**4. Gluteus medius in open chain side lying- Hip abductors**


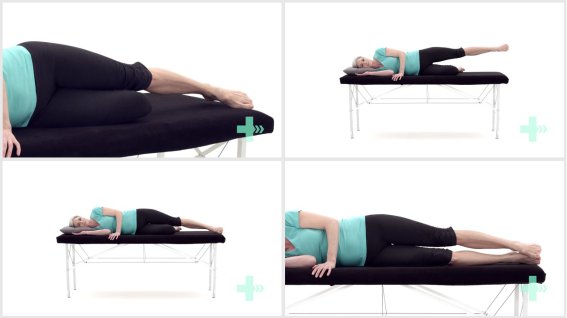


Lie on the bed on your side with your affected leg on top.

Bend your lower leg.

Keeping your upper leg straight, raise it towards the ceiling.

Hold for three seconds and then lower your leg back to the starting position.


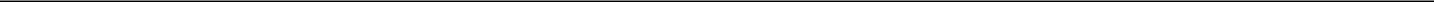


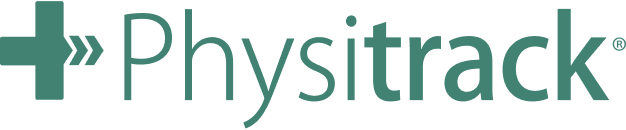


*Reprinted from****Physitrack PLC (***[*https://www.physitrack.com/*](https://gbr01.safelinks.protection.outlook.com/?url=https%3A%2F%2Fwww.physitrack.com%2F&data=05%7C02%7Canuj.punnoose%40nhs.net%7C6f900e1416ec45276ed308dc23e95b68%7C37c354b285b047f5b22207b48d774ee3%7C0%7C0%7C638424732439839895%7CUnknown%7CTWFpbGZsb3d8eyJWIjoiMC4wLjAwMDAiLCJQIjoiV2luMzIiLCJBTiI6Ik1haWwiLCJXVCI6Mn0%3D%7C0%7C%7C%7C&sdata=yOAYOJChg9BimlYLhg0VhbAREYYWlHeznaSpQOnog08%3D&reserved=0)***)****under a CC BY license, with permission from****Physitrack PLC****, original copyright****2012***


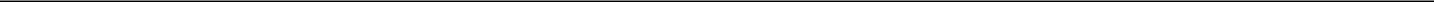
2 Sets / 10 Reps

**5. Standing active hip abduction- Hip abductors**


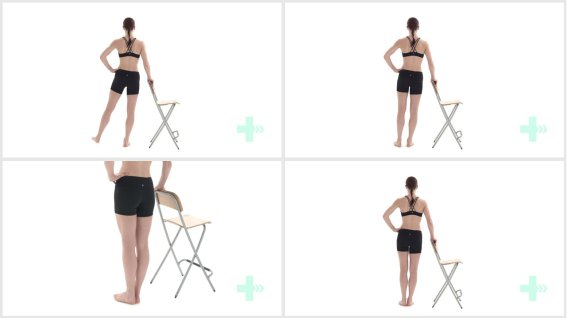


Stand straight, holding a chair or table for balance.

Keeping your affected leg straight, slowly move it out to the side.

Control the leg as you bring it back in to the starting position, and then repeat the movement.

Make sure you do not lean your body or hitch your hip up as you move your leg.


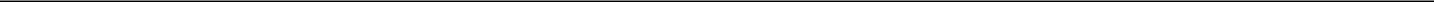


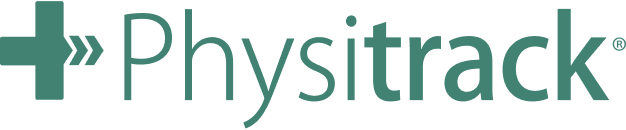


2 Sets / 10 Reps

**6. Step up- Hip abductors**


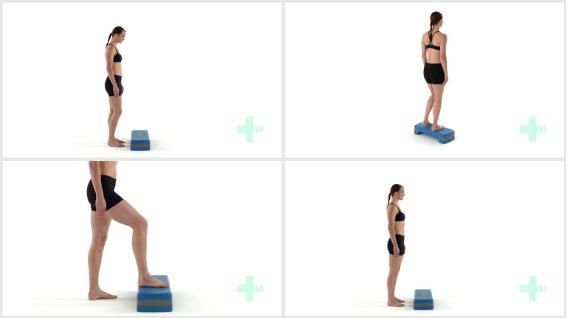


Stand facing a step.

Place your affected leg up on the step.

Step up bringing your other leg onto the step and then step back down to the start position using the same leg.

Make sure your knee travels forwards over your toes during this exercise.

Your affected leg will stay on the step throughout this exercise.


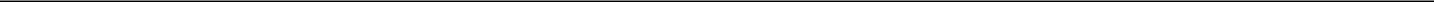


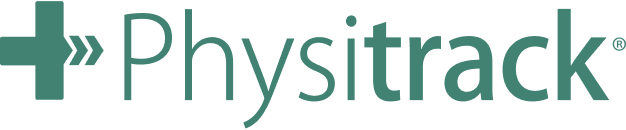


2 Sets / 10 Reps

**7. Sideways step up- Hip abductors**


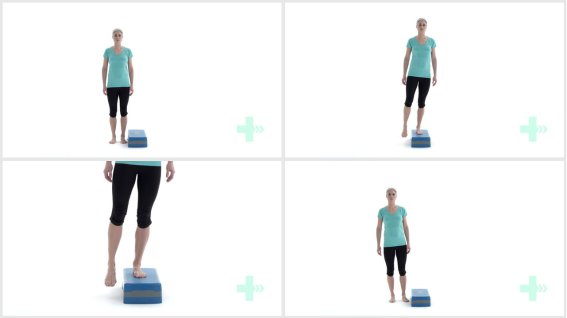


Stand up straight, sideways to a step.

Your affected leg should be closest to the step, and your feet close together.

You may use a handrail or chair for support if required.

Step your affected leg onto the step.

Step up, pushing through your affected leg, and stand tall, hovering your other leg above the floor.

Return your other leg to the floor, whilst keeping your affected leg on the step.


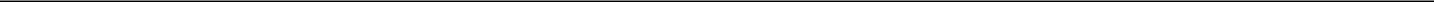


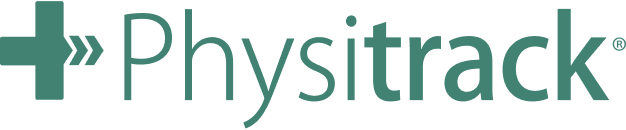


2 Sets / 10 Reps / 10 s hold

**8. Prone hip extension - leg bent- Hip extensors**


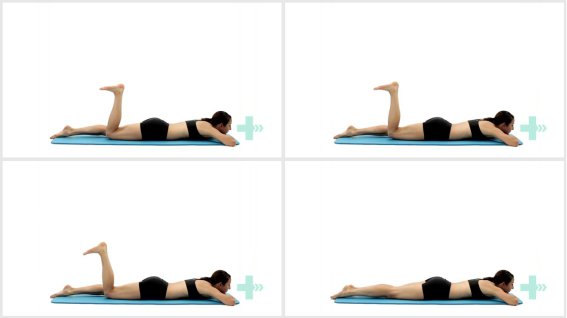


Lie on your tummy with your hands folded under your chin.

Bend the knee of your affected leg to a 90 degree angle, with your toes pulled up.

Push the heel towards the ceiling, squeezing your buttock muscles at all times.

Keep your hips flat on the floor.

Lower your leg back to the starting position and repeat.


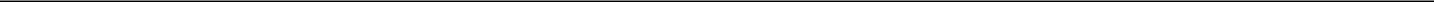


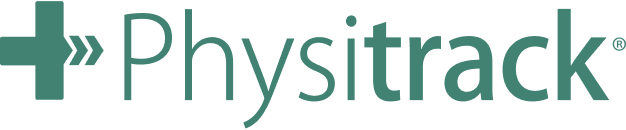


*Reprinted from****Physitrack PLC (***[*https://www.physitrack.com/*](https://gbr01.safelinks.protection.outlook.com/?url=https%3A%2F%2Fwww.physitrack.com%2F&data=05%7C02%7Canuj.punnoose%40nhs.net%7C6f900e1416ec45276ed308dc23e95b68%7C37c354b285b047f5b22207b48d774ee3%7C0%7C0%7C638424732439839895%7CUnknown%7CTWFpbGZsb3d8eyJWIjoiMC4wLjAwMDAiLCJQIjoiV2luMzIiLCJBTiI6Ik1haWwiLCJXVCI6Mn0%3D%7C0%7C%7C%7C&sdata=yOAYOJChg9BimlYLhg0VhbAREYYWlHeznaSpQOnog08%3D&reserved=0)***)****under a CC BY license, with permission from****Physitrack PLC****, original copyright****2012***


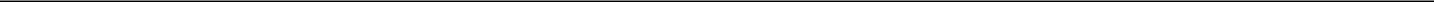
2 Sets / 10 Reps

**9. SL body hinge- Hip extensors**


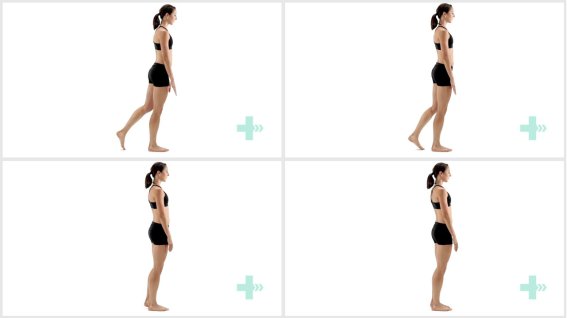


Stand on your affected leg with the opposite leg extended behind you. Tighten the abdominal and leg muscles as you hinge forward at the waist and raise the back leg.

Keep your body and leg in line with one another.


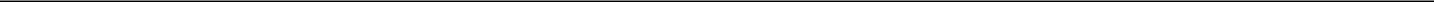


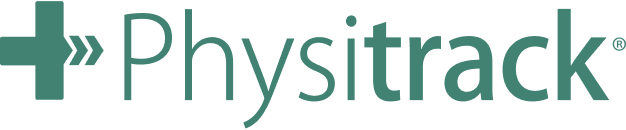


2 Sets / 10 Reps

**10. Pelvic floor - side-lying hip adduction- Hip adductors**


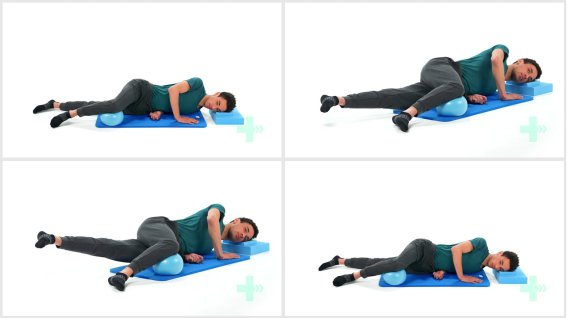


Lie on your side with your top leg bent and your bottom leg straight.

You can support the top leg with a towel or ball if you wish.

Squeeze and lift your pelvic floor as you lift the lower leg off the mat.

Relax your pelvic floor as you lower your leg back down.


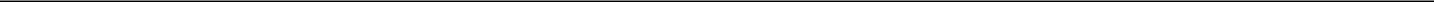


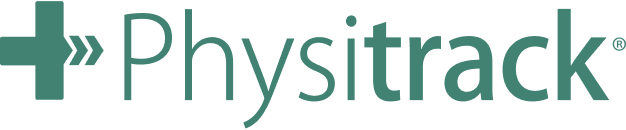


2 Sets / 10 Reps / 20 s hold

**11. Isometric hip adduction in supine (legs straight)- Hip adductors**


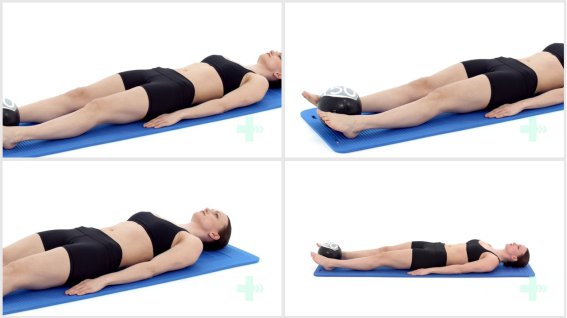


Lie on your back with a ball between your ankles.

Keeping your legs straight, tighten your abdominal muscles and squeeze the ball with your legs.

You should feel the muscles down the inside of your thighs tighten.

Hold this position, ensuring you breathe throughout.


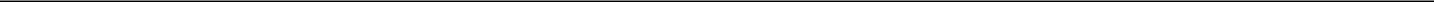


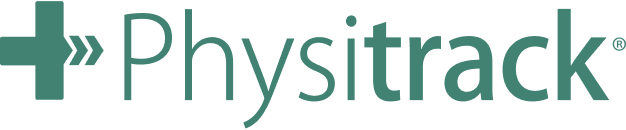


2 Sets / 10 Reps / 20 s hold

**12. Isometric hip adduction in crook lying- Hip adductors**


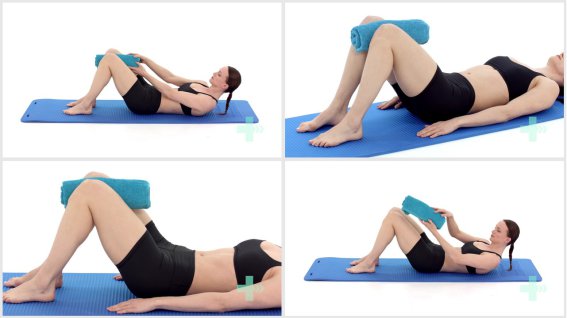


Lie on your back with your legs bent and feet flat on the floor.

Ensure your knees and feet are hips width apart.

Place a small ball or a rolled towel between your knees.

Tighten your abdominal muscles and pelvic floor, then squeeze your knees

together into the ball or towel.

Hold this position.

Relax, and then repeat.


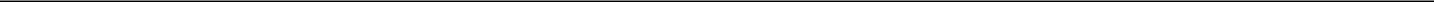


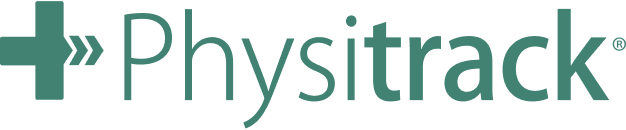


*Reprinted from****Physitrack PLC (***[*https://www.physitrack.com/*](https://gbr01.safelinks.protection.outlook.com/?url=https%3A%2F%2Fwww.physitrack.com%2F&data=05%7C02%7Canuj.punnoose%40nhs.net%7C6f900e1416ec45276ed308dc23e95b68%7C37c354b285b047f5b22207b48d774ee3%7C0%7C0%7C638424732439839895%7CUnknown%7CTWFpbGZsb3d8eyJWIjoiMC4wLjAwMDAiLCJQIjoiV2luMzIiLCJBTiI6Ik1haWwiLCJXVCI6Mn0%3D%7C0%7C%7C%7C&sdata=yOAYOJChg9BimlYLhg0VhbAREYYWlHeznaSpQOnog08%3D&reserved=0)***)****under a CC BY license, with permission from****Physitrack PLC****, original copyright****2012***


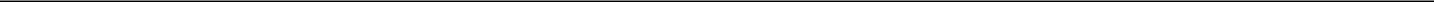
2 Sets / 10 Reps

**13. Standing hip abduction/adduction slide-Hip abductors/adductors**


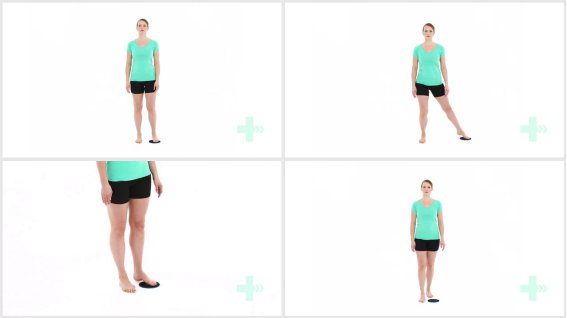


Stand up straight on a polished floor.

Place a paper towel or slider under one foot.

Use shoes with a good grip if required.

Keep your posture up straight and your feet pointing forward.

Tighten your abdominal muscles and slide your foot on the paper along the floor out to the side.

Ensure you keep weight on this leg by taking your body with the movement.

Focus as you slide the foot along the floor back in towards your stationary leg.

You should feel the muscles in your inner thigh tense as you do this.


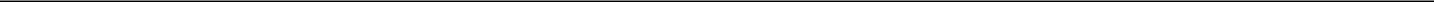


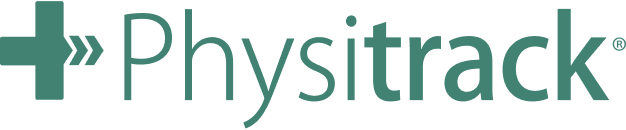


2 Sets / 10 Reps

**14. Resisted kneeling hip external rotation- Hip external rotators**


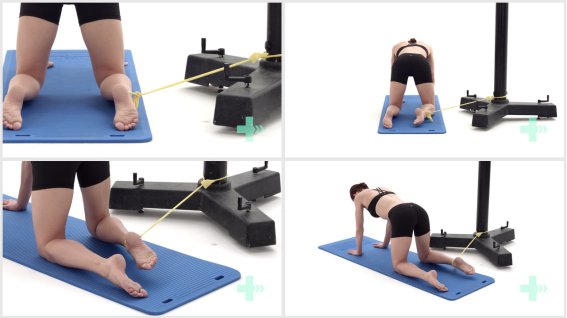


Start on your hands and knees, with your hands under your shoulders and knees under your hips.

Tie a resistance band around the heel on your affected side.

Secure it around a sturdy object.

Keeping your hands and knees in the same position, pull your foot attached to the resistance band inwards, pivoting around your knee. You should feel an effort pulling against the band.

Control the movement as you move your foot back to the starting position.


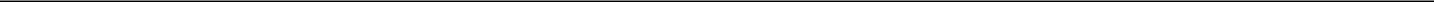


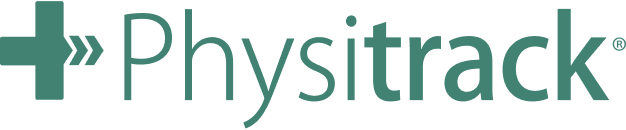


2 Sets / 10 Reps

**15. Resisted hip external rotation in semi-squat- Hip external rotators**


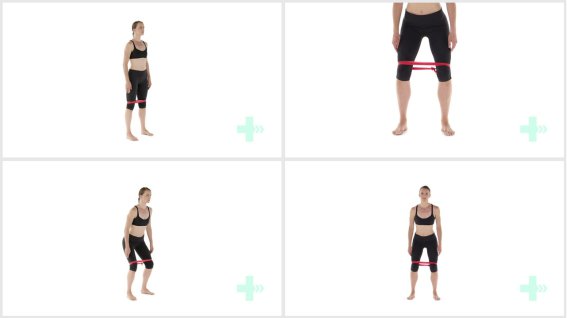


Stand up straight with a resistance band tied around both thighs, just above your knees.

Step your feet hip-distance apart.

Ensure there is tension in the band.

Keeping your back straight, push your hips back and bend your knees a little. Maintaining this position push your knees outwards against the resistance of the band.

Control the movement as you return your knees to the starting position, and then repeat.


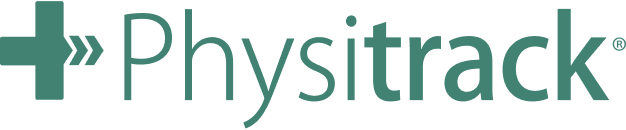


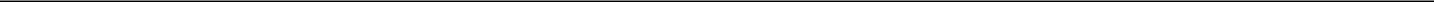


2 Sets / 10 Reps

**16. Bilateral knee fall out- Core muscles**


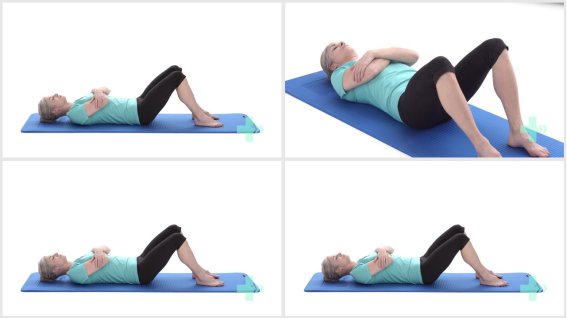


Lie on your back with your knees bent and your feet flat on the floor.

Initiate your core stability muscles and slowly drop your knees out to the sides. Control the movement as you bring your knees back up to the starting position. Ensure you keep your trunk and pelvis still and do not allow your lower back to arch.


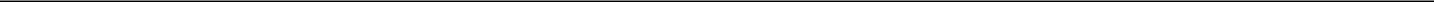


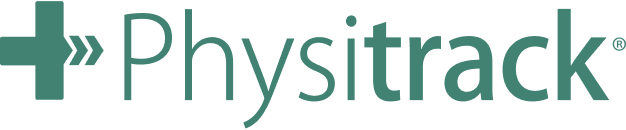


*Reprinted from****Physitrack PLC (***[*https://www.physitrack.com/*](https://gbr01.safelinks.protection.outlook.com/?url=https%3A%2F%2Fwww.physitrack.com%2F&data=05%7C02%7Canuj.punnoose%40nhs.net%7C6f900e1416ec45276ed308dc23e95b68%7C37c354b285b047f5b22207b48d774ee3%7C0%7C0%7C638424732439839895%7CUnknown%7CTWFpbGZsb3d8eyJWIjoiMC4wLjAwMDAiLCJQIjoiV2luMzIiLCJBTiI6Ik1haWwiLCJXVCI6Mn0%3D%7C0%7C%7C%7C&sdata=yOAYOJChg9BimlYLhg0VhbAREYYWlHeznaSpQOnog08%3D&reserved=0)***)****under a CC BY license, with permission from****Physitrack PLC****, original copyright****2012***


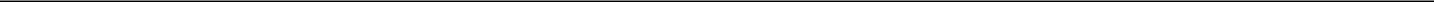
1 Set / 10 Reps / 5 s hold

**17. Side plank on knees- Trunk +Core**


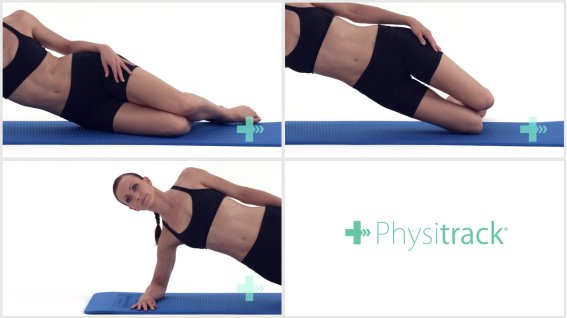


Lie on your side and prop yourself up on your elbow.

Bend your knees and lift your hips off the mat until you have a straight line from

your knees to the top of your head.

Hold this position for as long as you can.

*Perform on both sides*


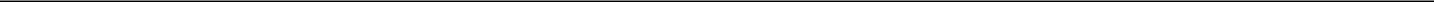


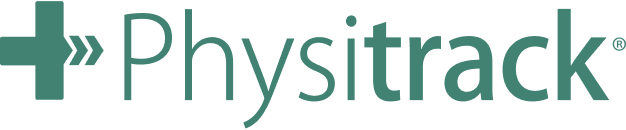


2 Sets / 10 Reps

**18. Bird dog- Trunk**


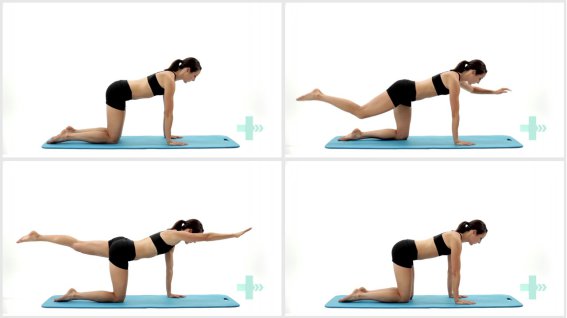


Start on your hands and knees, with your hands under your shoulders, and knees under your hips.

Tighten the abdominal core muscles.

Extend the opposite leg and the opposite arm simultaneously, making sure your maintain good control in your torso.

Do not allow your body or hips to rotate.

Repeat on the other side.


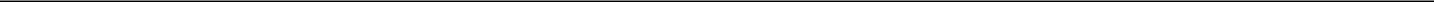


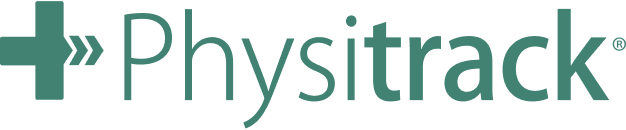


2 Sets / 10 Reps / 10 s hold

**19. SLS on foam balance mat- Proprioception**


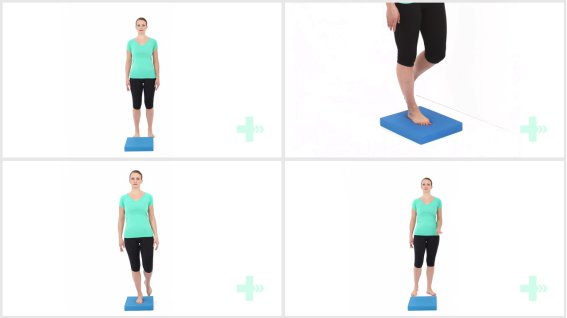


Stand up straight close to a wall or solid supporting surface.

Place a foam balance mat on the floor in front of you.

Step onto the balance mat with your affected foot, using the wall to balance as needed.

Try to let go of the wall while keeping your balance.

Ensure you keep your gaze straight ahead, and your thigh and buttock muscles tightened.

Hold this position.


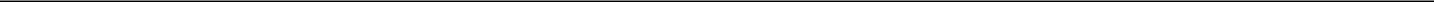


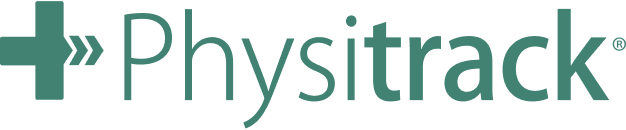


2 Sets / 5 Reps / 10 sec duration

**20. Single-leg balance on Bosu- Proprioception**


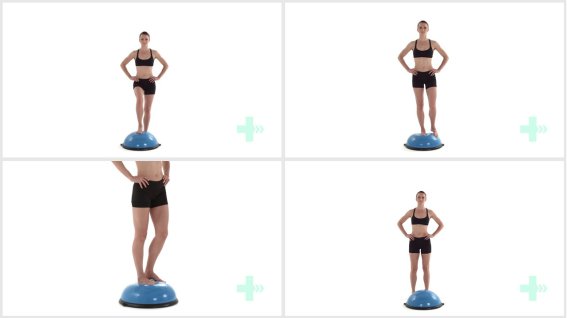


Place a Bosu ball on the floor on the flat side near a wall so you can hold on for balance if needed.

Stand on your affected leg on the centre of the Bosu, and let go.

Try and balance here for as long as you can.

You can make this exercise more difficult by turning your head, or by closing your eyes.


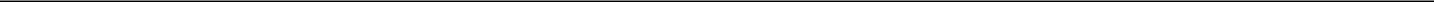


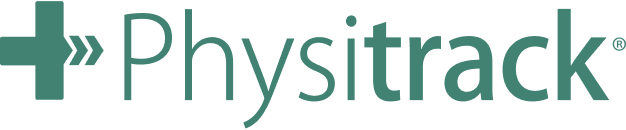


*Reprinted from****Physitrack PLC (***[*https://www.physitrack.com/*](https://gbr01.safelinks.protection.outlook.com/?url=https%3A%2F%2Fwww.physitrack.com%2F&data=05%7C02%7Canuj.punnoose%40nhs.net%7C6f900e1416ec45276ed308dc23e95b68%7C37c354b285b047f5b22207b48d774ee3%7C0%7C0%7C638424732439839895%7CUnknown%7CTWFpbGZsb3d8eyJWIjoiMC4wLjAwMDAiLCJQIjoiV2luMzIiLCJBTiI6Ik1haWwiLCJXVCI6Mn0%3D%7C0%7C%7C%7C&sdata=yOAYOJChg9BimlYLhg0VhbAREYYWlHeznaSpQOnog08%3D&reserved=0)***)****under a CC BY license, with permission from****Physitrack PLC****, original copyright****2012***

**Prehabilitation for FAI surgery- Phase** **2 (5-8 weeks)**


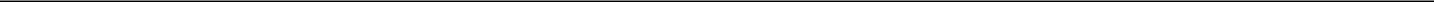


3 Sets / 1 Rep / 15 min duration / 100 bpm / 6 RPE

**1. Stationary cycle spin bike- Cardiovascular fitness**


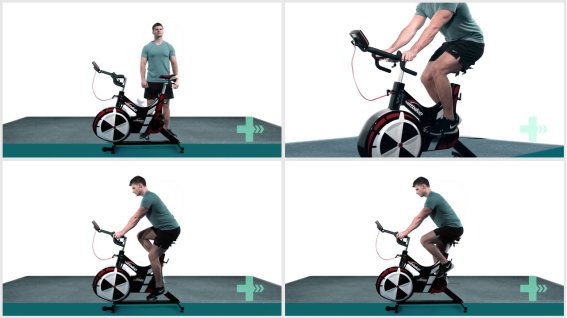


Adjust the bike seat and settings as required and commence cycling.

*Aim for 150 minutes of moderate exercise per week -Swimming, Cycling, Flat terrain walking, Elliptical cross trainer*


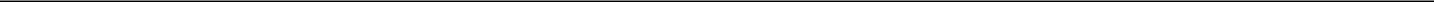


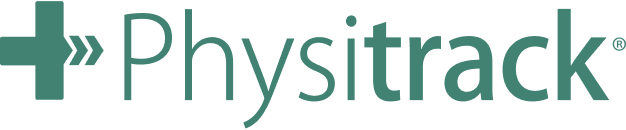


2 Sets / 10 Reps / 5 s hold

**2. Bridge single leg- All gluteals**


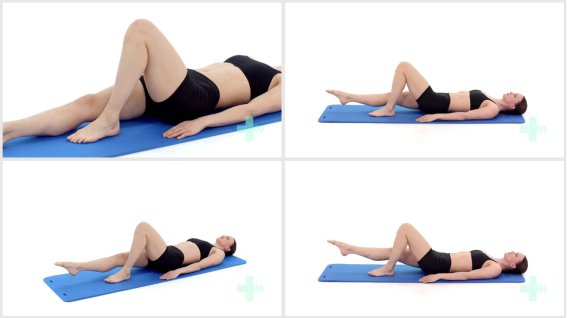


Lie on your back with your legs hips width apart.

Bend the leg you want to strengthen, keeping your foot on the floor.

Your knee and foot should still be in alignment with your hip.

Tighten your abdominal and buttock muscles and lift your hips up off the floor, allowing your straight leg to lift with the movement. Your thighs should remain level.

Ensure your hips do not drop on the side of your straight leg.

Control the movement as you lower your hips back down to the floor and repeat the movement.


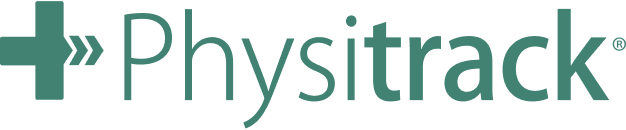


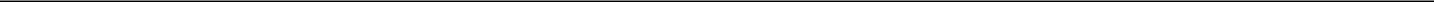


2 Sets / 10 Reps

**3. Pelvic drop- Hip abductors**


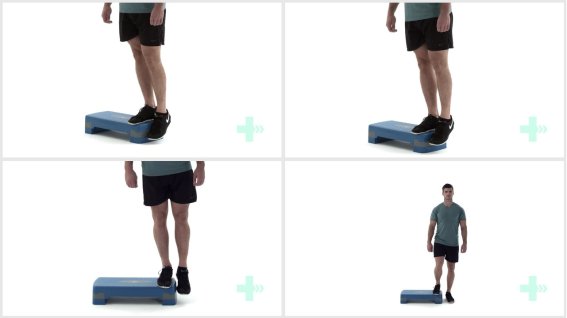


Stand with your affected leg on a stair or step.

Let your other foot drop slowly to the floor sideways, but do not flex your knee or hip in the movement.

Move in a slow and controlled pace during this exercise.

Move your leg back into the starting position and repeat.


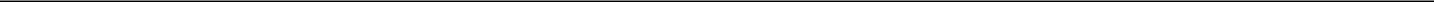


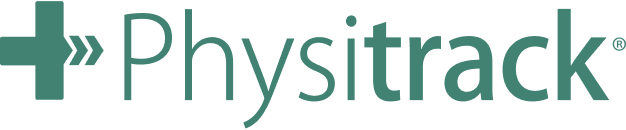


2 Sets / 10 Reps

**4. Resisted hip abduction in side lying (resistance at ankles)- Hip abductors**


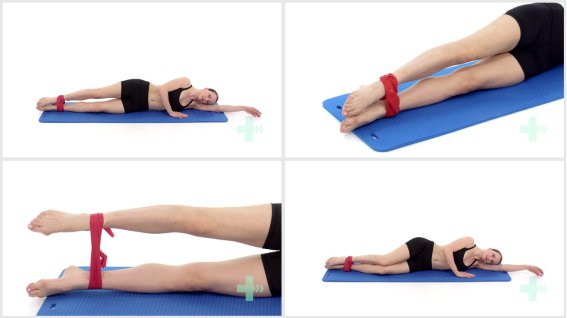


Lie on your side with your affected leg on top.

Tie a resistance band around both of your ankle.

Keeping your top leg straight, lift it directly upwards pulling against the resistance of the band.

Slowly lower your leg back down controlling the movement against the band.

Relax and repeat.


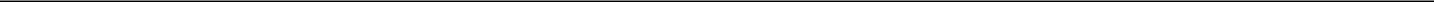


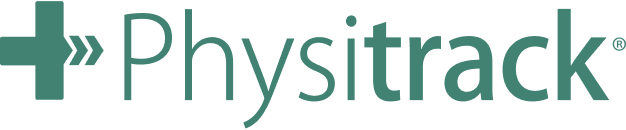


*Reprinted from****Physitrack PLC (***[*https://www.physitrack.com/*](https://gbr01.safelinks.protection.outlook.com/?url=https%3A%2F%2Fwww.physitrack.com%2F&data=05%7C02%7Canuj.punnoose%40nhs.net%7C6f900e1416ec45276ed308dc23e95b68%7C37c354b285b047f5b22207b48d774ee3%7C0%7C0%7C638424732439839895%7CUnknown%7CTWFpbGZsb3d8eyJWIjoiMC4wLjAwMDAiLCJQIjoiV2luMzIiLCJBTiI6Ik1haWwiLCJXVCI6Mn0%3D%7C0%7C%7C%7C&sdata=yOAYOJChg9BimlYLhg0VhbAREYYWlHeznaSpQOnog08%3D&reserved=0)***)****under a CC BY license, with permission from****Physitrack PLC****, original copyright****2012***


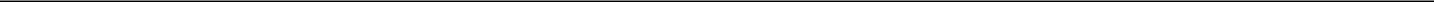
2 Sets / 10 Reps / 5 s hold

**5. Resisted hip abduction (standing with post)- Hip abductors**


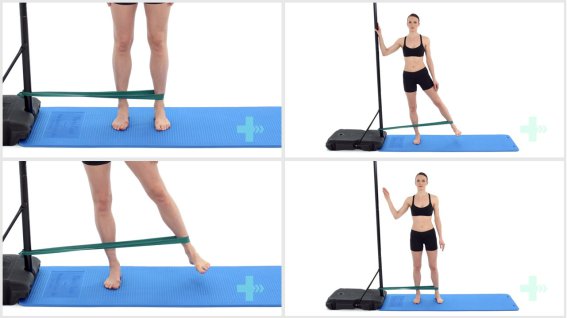


Stand up straight with a looped resistance band tied to a solid object at one side.

Step the leg furthest away from the band into the loop.

This will be the leg you exercise.

Stand with your legs hips width apart and some slight tension in the band. Keeping your back straight and your hips level, lift your leg with the band attached out to one side.

Ensure you do not lean your body or hitch your hip.

Your leg should remain straight and move directly out to the side.

Control the movement as you bring your leg back in to the starting position.


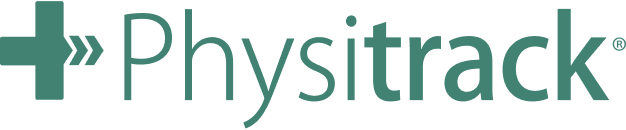


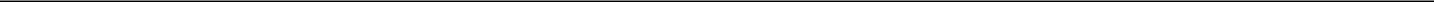


2 Sets / 10 Reps / 5kg weight

**6. Stiff leg deadlift - single arm, single leg with dumbbell- Hip extensors**


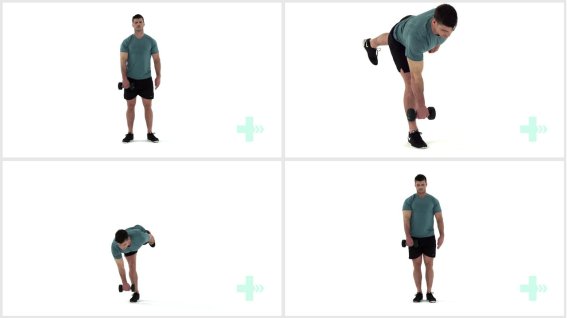


Stand with your knees slightly bent, holding a dumbbell in one hand. Hinge forward at the waist and fully extend the opposite leg out to the rear, lowering the dumbbell towards your shin.

Keep your back straight and do not allow your body to rotate.

Contract your gluteus and hamstrings and return to a standing position.


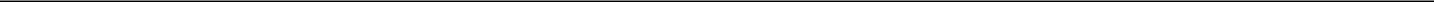


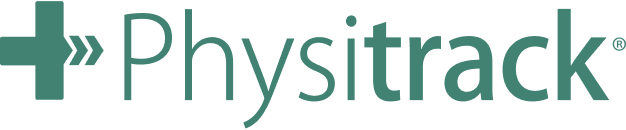


2 Sets / 10 Reps / 5 s hold

**7. Modified Plank with hip extension- Hip extensors**


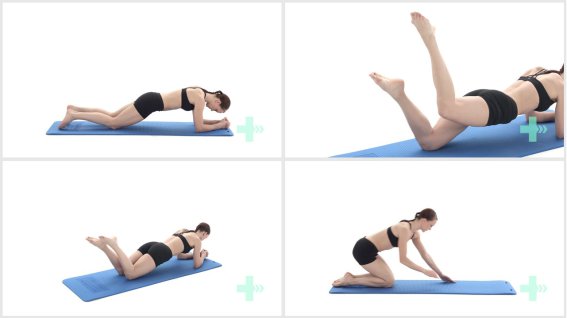


Lie on your front with both knees bent.

Lift your body up onto your forearms, ensuring your elbows are under your shoulders.

There should only be a slight angle at your hips.

Holding this position, lift the knee of your affected leg off the floor, pushing the sole of your foot up towards the ceiling.

Hold this position before you lower it back down to the starting position, and then repeat.


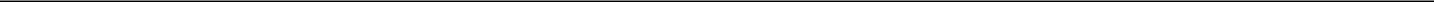


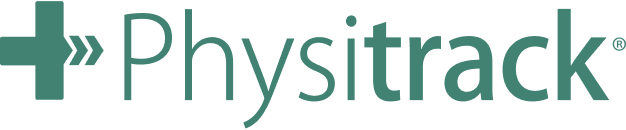


2 Sets / 10 Reps / 5 s hold

**8. Plank with hip extension- Hip extensors**


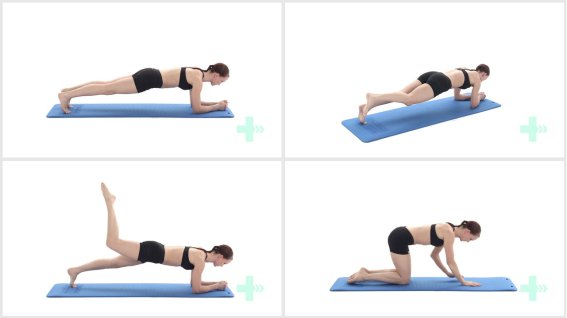


Lie on your front and lift yourself up into a plank position.

Your forearms should be on the floor with your elbows under your shoulders, and your toes should be tucked under.

You should endeavour to hold a straight line from the tip of your head to your heels.

Hold this position as you bend one knee, then extend this hip, pushing your foot up towards the ceiling.

Keep a hips width gap between your legs.

Lower this leg back to the plank position, and repeat.


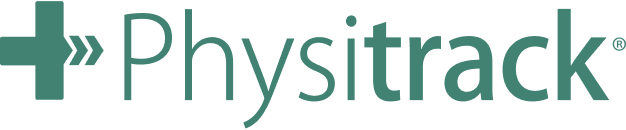


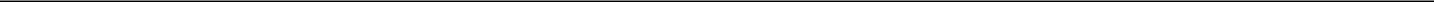


*Reprinted from****Physitrack PLC (***[*https://www.physitrack.com/*](https://gbr01.safelinks.protection.outlook.com/?url=https%3A%2F%2Fwww.physitrack.com%2F&data=05%7C02%7Canuj.punnoose%40nhs.net%7C6f900e1416ec45276ed308dc23e95b68%7C37c354b285b047f5b22207b48d774ee3%7C0%7C0%7C638424732439839895%7CUnknown%7CTWFpbGZsb3d8eyJWIjoiMC4wLjAwMDAiLCJQIjoiV2luMzIiLCJBTiI6Ik1haWwiLCJXVCI6Mn0%3D%7C0%7C%7C%7C&sdata=yOAYOJChg9BimlYLhg0VhbAREYYWlHeznaSpQOnog08%3D&reserved=0)***)****under a CC BY license, with permission from****Physitrack PLC****, original copyright****2012***


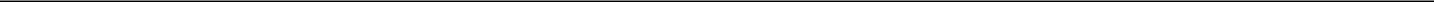
2 Sets / 10 Reps

**9. Resisted hip adduction (standing with post)- Hip adductors**


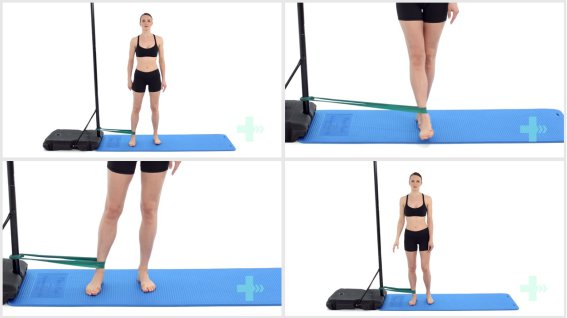


-

Stand up straight with a looped resistance band tied to a solid object at one side.

Step the leg nearest the band into the loop.

This will be the leg you exercise.

Lift the leg with the band out to the side ensuring there is still some slight tension in the band.

Keeping your back straight and your hips level, move your leg with the band attached straight across in front of your stance leg.

You may only manage to bring your leg to meet your stance leg.

Ensure you do not lean your body or hitch your hip.


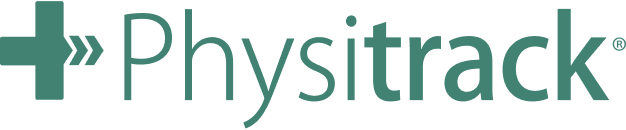


Your leg should remain straight.

Control the movement as you bring your leg back out to the starting position.


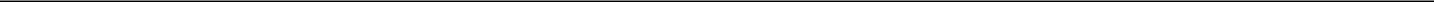


2 Sets / 10 Reps / 5 s hold

**10. Copenhagen hip adduction exercise- Hip adductors**


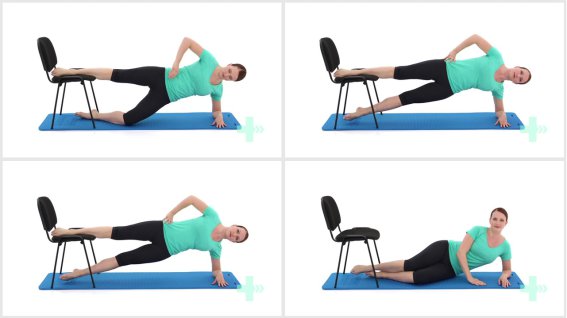


Lie on your side with the leg you would like to exercise on top.

Your upper body is supported on your forearm with your elbow straight under your shoulder.

Place the foot of the top leg on a sturdy platform, such as a chair.

Lift your hips up so that you have a straight line from your body through to your elevated leg.

Hold this position while lifting your lower leg up towards the elevated leg and lowering it back down.


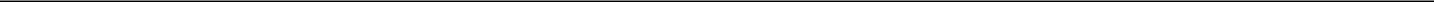


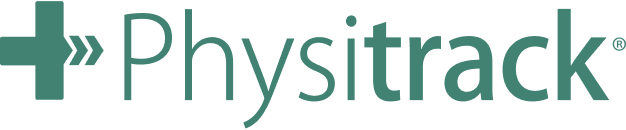


2 Sets / 10 Reps

**11. Resisted hip external rotation in prone- Hip external rotators**


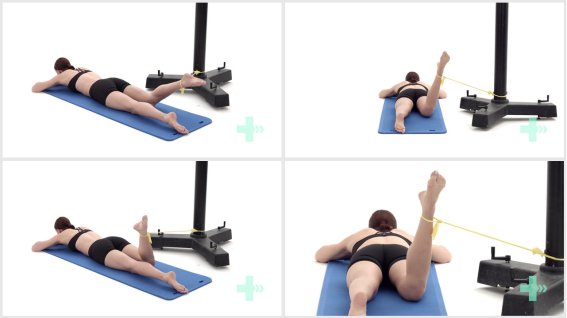


Lie on your front with a resistance band tied around the ankle on your affected side.

Secure the band around a sturdy object.

Bend the knee on your affected side to 90 degrees and allow the lower leg to rotate outwards with the pull of the band.

Keep your thighs close together and rotate the lower leg inwards, pulling against the band.

Ensure your knee stays at 90 degrees.

Control the movement as you rotate the lower leg back outwards.


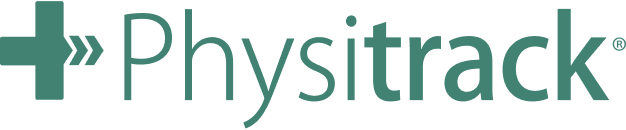


1 Set / 10 Reps / 5 s hold

**12. Side plank- Trunk+Core**

Lie on your side, propping yourself up on your elbow.

Keep your legs straight and stacked on top of one another.

Use your elbow and feet to push the body off the floor, and maintain a straight line from your head to your feet.

Hold this position for as long as you can, preventing the hips from sagging.

*Reprinted from****Physitrack PLC (***[*https://www.physitrack.com/*](https://gbr01.safelinks.protection.outlook.com/?url=https%3A%2F%2Fwww.physitrack.com%2F&data=05%7C02%7Canuj.punnoose%40nhs.net%7C6f900e1416ec45276ed308dc23e95b68%7C37c354b285b047f5b22207b48d774ee3%7C0%7C0%7C638424732439839895%7CUnknown%7CTWFpbGZsb3d8eyJWIjoiMC4wLjAwMDAiLCJQIjoiV2luMzIiLCJBTiI6Ik1haWwiLCJXVCI6Mn0%3D%7C0%7C%7C%7C&sdata=yOAYOJChg9BimlYLhg0VhbAREYYWlHeznaSpQOnog08%3D&reserved=0)***)****under a CC BY license, with permission from****Physitrack PLC****, original copyright****2012***

1 Set / 10 Reps / 5 s hold

**13. Side plank with trunk twist- Trunk +Core**

Lie on your side with your legs straight.

Place your top foot in front of your bottom foot on the floor.

Lift yourself up on your side using the lower arm with the hand on the floor.

Your hips will come up off the floor until your body is a straight line from your head to your feet.

Lift your upper arm directly up so that it is in a vertical position.

Maintaining control with your body, bring your upper arm back down and around and under the gap between your body and the floor.

You will rotate round with this movement but do not allow your body to sink or wobble too much.

Immediately return back to the start position and repeat.

2 Sets / 5 Reps

**14. SLS dynamic balance cones push- Proprioception**

Stand up straight and place four cones closely around your affected foot.

One in front, one behind, one on the inside and one on the outside.

Transfer your weight onto your affected foot and lift your opposite leg off the floor. Whilst keeping your balance, use your non affected foot to push the cones as far away from you as you can.

Next, use your non affected foot to pull the cones back to the start position.

2 Sets / 5 Reps

**15. Squat on BOSU (uneven side)- Proprioception**

Place a BOSU ball on the floor on the flat side.

Step up onto the BOSU, standing on the soft side with your feet hips width apart and toes pointing forwards.

Tightening your abdominal muscles and keeping your back straight, bend your knees lowering down into a squat.

Ensure your knees travel directly forwards over your outer toes.

Keep your gaze ahead.

Control the movement as you straighten back up again.

*Reprinted from****Physitrack PLC (***[*https://www.physitrack.com/*](https://gbr01.safelinks.protection.outlook.com/?url=https%3A%2F%2Fwww.physitrack.com%2F&data=05%7C02%7Canuj.punnoose%40nhs.net%7C6f900e1416ec45276ed308dc23e95b68%7C37c354b285b047f5b22207b48d774ee3%7C0%7C0%7C638424732439839895%7CUnknown%7CTWFpbGZsb3d8eyJWIjoiMC4wLjAwMDAiLCJQIjoiV2luMzIiLCJBTiI6Ik1haWwiLCJXVCI6Mn0%3D%7C0%7C%7C%7C&sdata=yOAYOJChg9BimlYLhg0VhbAREYYWlHeznaSpQOnog08%3D&reserved=0)***)****under a CC BY license, with permission from****Physitrack PLC****, original copyright****2012***

**Prehabilitation for FAI surgery-** **Manual therapy and stretching**

1 Set / 10 Reps / 10 s hold

**1. Psoas muscle stretch**

Kneel down on one knee.

Ensure your rear knee is directly under your hip and your front knee is in line with your front heel.

Now tuck your bottom under, opening out through the front of the hip you are kneeling on.

To increase the stretch, gently lean to the opposite side and place the arm of the same side you are stretching up above your head and stretch towards the ceiling. Maintain the position for 30-60 seconds.

1 Set / 10 Reps / 10 sec duration

**2. Self trigger point mobilisation to ITB with dynamic movement**

Sit up straight with your legs hanging over the edge of a bed.

Place your hand over the end of your thigh, applying a deep pressure to the structures on the side of your knee.

When you feel a tender spot, hold this position and then slowly straighten and bend your knee.

Continue this fluid movement until you feel the tissues release.

Finish up with some gentle circular sweeps of the tissues around your knee.

1 Set / 10 Reps / 10 sec duration

**3. Gluteal self massage and trigger point with spiky-ball supine**

Place the spiky-ball on the floor and position your affected buttock on the ball.

Using your arms, move your buttock over the ball.

You can vary the amount of pressure through your gluteal region by changing the amount of weight you place through your arms.

When you find a particularly tender area, hold this position, increasing the pressure through the ball.

1 Set / 10 Reps / 10 sec duration

**4. Lateral gluteal self massage and trigger point with spiky-ball**

Place the spiky-ball on the floor and lie on your affected side.

Position the side of your affected buttock on the ball.

Using your arms, move your buttock over the ball.

You can vary the amount of pressure through your gluteal region by changing the amount of weight you place through your arms.

When you find a particularly tender area, hold this position, increasing the pressure through the ball.

*Reprinted from****Physitrack PLC (***[*https://www.physitrack.com/*](https://gbr01.safelinks.protection.outlook.com/?url=https%3A%2F%2Fwww.physitrack.com%2F&data=05%7C02%7Canuj.punnoose%40nhs.net%7C6f900e1416ec45276ed308dc23e95b68%7C37c354b285b047f5b22207b48d774ee3%7C0%7C0%7C638424732439839895%7CUnknown%7CTWFpbGZsb3d8eyJWIjoiMC4wLjAwMDAiLCJQIjoiV2luMzIiLCJBTiI6Ik1haWwiLCJXVCI6Mn0%3D%7C0%7C%7C%7C&sdata=yOAYOJChg9BimlYLhg0VhbAREYYWlHeznaSpQOnog08%3D&reserved=0)***)****under a CC BY license, with permission from****Physitrack PLC****, original copyright****2012***

1 Set / 5 Reps / 10 sec duration

**5. Glute myofascial release with ball**

Place a ball on the floor.

Cross the leg to be stretched over the opposite knee and place the ball under your buttock.

Roll over the ball until you reach a tender point deep in the gluteus muscles, and then hold this position.

1 Set / 10 Reps / 3 s hold

**6. Lateral hip self mobilisation**

Kneel up straight.

Tie a resistance band around your affected hip, ensuring it lies close to the joint.

Tie the other ends of the band to a secure object such as a pole or door handle.

Bring your affected leg forwards so that your foot is flat on the floor.

Move forwards until you feel a stretch in your hip.

Hold this position and rock forwards and backwards rhythmically.

You should feel a gentle outward pulling on your hip from the band.

1 Set / 10 Reps / 10 s hold

**7. Piriformis stretch**

Lie on your back and bend your affected knee.

Cross this leg over your other knee, placing the outside of your ankle just above the knee of your good leg.

Let the knee on your affected leg drop out to the side, and bend the good leg, sliding your heel towards your buttocks.

You may feel a stretch through your affected buttock.

To increase this stretch, interlace your fingers behind the thigh of your good leg, and pull your thigh in towards you, lifting the foot off the ground.

To increase this stretch further still, push your elbow into the thigh of your affected leg.

Hold this position.

*Reprinted from****Physitrack PLC (***[*https://www.physitrack.com/*](https://gbr01.safelinks.protection.outlook.com/?url=https%3A%2F%2Fwww.physitrack.com%2F&data=05%7C02%7Canuj.punnoose%40nhs.net%7C6f900e1416ec45276ed308dc23e95b68%7C37c354b285b047f5b22207b48d774ee3%7C0%7C0%7C638424732439839895%7CUnknown%7CTWFpbGZsb3d8eyJWIjoiMC4wLjAwMDAiLCJQIjoiV2luMzIiLCJBTiI6Ik1haWwiLCJXVCI6Mn0%3D%7C0%7C%7C%7C&sdata=yOAYOJChg9BimlYLhg0VhbAREYYWlHeznaSpQOnog08%3D&reserved=0)***)****under a CC BY license, with permission from****Physitrack PLC****, original copyright****2012***
