# Supplementary material for: PREHAB FAI- Prehabilitation for patients undergoing arthroscopic hip surgery for Femoroacetabular Impingement Syndrome -Protocol for an assessor blinded randomised controlled feasibility study
Source: PLoS One. 2024 Apr 11;19(4):e0301194. doi: 10.1371/journal.pone.0301194 (PMC11008823; doi:10.1371/journal.pone.0301194)
Supplement: S2 Appendix — (DOCX) [file pone.0301194.s002.docx]

**S2 Appendix. Sample consent form**

Study Number: A096465, IRAS Project ID: 293927

Patient Identification Number/ CRN for this trial:

**CONSENT FORM**

**Title of Study:** Prehabilitation for patients undergoing arthroscopic hip surgery for Femoroacetabular Impingement Syndrome- A feasibility study

**Name of Researcher:**  Please initial box

1. I confirm that I have read and understand the Patient Information Sheet,

version x, dated xxxx, for the above study and have had the

opportunity to ask questions.

2. I understand that my participation is voluntary and that I am free to withdraw

at any time, without giving any reason, without my medical care or legal rights

being affected.

3. I understand that sections of my medical notes and data collected

during the study may be looked at by responsible individuals from the

research team, regulatory authorities, sponsor or from the NHS Trust,

where it is relevant to the patient taking part in this study. I give permission

for these individuals to have access to the patient’s records.

4. I give consent to participate in this study and allow my data to be stored in the

research study database and shared with the research team.

_______________________ _____________ __________________

Name Participant Date Signature

_______________________ _____________ __________________

Name of Person taking consent Date Signature

(if different from researcher)

_______________________ _____________ __________________

Researcher Date Signature

**1 copy for participant, 1 copy for research team.**
