# Supplementary material for: PREHAB FAI- Prehabilitation for patients undergoing arthroscopic hip surgery for Femoroacetabular Impingement Syndrome -Protocol for an assessor blinded randomised controlled feasibility study
Source: PLoS One. 2024 Apr 11;19(4):e0301194. doi: 10.1371/journal.pone.0301194 (PMC11008823; doi:10.1371/journal.pone.0301194)
Supplement: S3 Appendix — (DOCX) [file pone.0301194.s003.docx]

**S3 Appendix. Objective outcome measurements**

**Hip muscle strength tests [6, 46]**

All hip strength tests (Isometric) will be done using a Handheld dynamometer (HHD). Each strength test will be performed 3 times- 2 reps with submaximal force and then the 3^rd^ attempt as hard as possible and hold for 5 seconds. The highest value from the three will be considered as the final result. Rest time of 5 seconds will be allowed between each repetition and 30 seconds minimum between each test. An external belt will be used to fix the HHD to improve inter-rater reliability [46].

**Supine position**

**Abduction strength [49]**

Test leg resting in neutral

Participant will be asked to hold onto the exam table to stabilise the trunk

HHD will be placed 5 cm above the lateral malleolus of the testing limb and fixed with an external belt attached to a handle fixed onto the wall.

Instruction: ‘Go ahead, push, push, push and relax’.

**Adduction strength [49]**

Test leg resting in neutral

Participant will be asked to hold onto the exam table to stabilise the trunk

HHD will be placed 5 cm above the medial malleolus of the testing limb and fixed with an external belt attached to a handle fixed onto the wall.

Instruction: ‘Go ahead, push, push, push and relax’.

**Prone position**

**Extension strength [6]**

Test leg knee bend at 90 deg and placed on the edge of the foot of the exam table

Participant will be asked to hold onto the exam table to stabilise the trunk

HHD will be placed over the centre of the heel of the testing limb and fixed with an external belt attached to a handle fixed on the floor.

Instruction: ‘Go ahead, push the foot towards the ceiling, push, push, push and relax’.

**Sitting**

**Flexion strength [49]**

Participant sitting on the edge of the exam table with knee flexed at 90 deg

Participant will be asked to hold onto the exam table with both hands to stabilise the body

HHD placed 5cm above the superior pole of the patella and fixed with an external belt attached to a handle fixed on the floor.

Instruction: ‘Go ahead, pull your knee up to the ceiling, pull, pull, pull and relax’

**Internal rotation strength [49]**

Participant sitting on the edge of the exam table with both knees flexed at 90 deg

Participant will be asked to hold onto the exam table with both hands to stabilise the body

HHD placed 5cm above the lateral malleolus fixed with an external belt attached to a handle fixed on the floor.

Instruction: ‘Go ahead, push, push, push and relax’

**External rotation strength [49]**

Participant sitting on the edge of the exam table with both knees flexed at 90 deg

Participant will be asked to hold onto the exam table with both hands to stabilise the body

HHD placed 5cm above the medial malleolus and fixed with an external belt attached to a handle fixed on the floor.

Instruction: ‘Go ahead, push, push, push and relax’

**Side lying**

**Trunk endurance [80]**

Participant will be in the side lying position with the hip resting on the exam table or the floor mat and one leg resting over the other.

Participant will be instructed to lift the hip off the floor and hold the position for as long as possible supporting the weight through the forearm and feet.

The time (seconds) will be recorded from the start until the end of the test when the participant’s hip touches the floor.

Instruction: ‘Go on, lift your hip off the floor and hold the position as much as possible’.

Verbal prompts will be given to the patient every 30 sec.

**Functional Test**

**Star Excursion Balance Test (SEBT)[6, 51]**

Anterior

Anteromedial

Anterolateral

Lateral

Medial

Posterolateral

Posteromedial

Posterior

**Fig 1. SEBT directions for left leg stance**

We will measure 4 different directions namely anterior, anteromedial, posteromedial and posterolateral.

The participant will be asked to stand on one leg at the centre of the circle. While maintaining the stance, they will be asked to reach with the other foot as far as possible and touch the line with their big toe. The test will be performed starting from the anterior direction clockwise. The assessor will measure the distance in all four directions. The test will be invalid if the participant (i) failed to maintain stance, (ii) lifts or moves the stance foot from the centre point, (iii) touches the reach foot down fully and (iv) Unable to bring the reach foot back to the starting position.

The participant will be allowed a trial attempt on either leg and test will be conducted on the affected side.
