# Supplementary material for: PREHAB FAI- Prehabilitation for patients undergoing arthroscopic hip surgery for Femoroacetabular Impingement Syndrome -Protocol for an assessor blinded randomised controlled feasibility study
Source: PLoS One. 2024 Apr 11;19(4):e0301194. doi: 10.1371/journal.pone.0301194 (PMC11008823; doi:10.1371/journal.pone.0301194)
Supplement: S1 File — (DOCX) [file pone.0301194.s005.docx]

**RESEARCH PROTOCOL**

**Title**

**PREHAB FAI**- Prehabilitation for patients undergoing arthroscopic hip surgery for Femoro-acetabular impingement syndrome - Protocol for an assessor blinded randomised controlled feasibility study

**Chief Investigator & Supervisor**: Mr Vikas Khanduja, MA (Cantab), MSc, FRCS (Tr& Orth), PhD, Addenbrooke’s – Cambridge University Hospital, Cambridge, UK

**Principal Investigator**: Mr Anuj Punnoose, MSc, MCSP, Addenbrooke’s – Cambridge University Hospital, Cambridge, UK

**Collaborators & PhD Supervisors**:

Dr Leica Claydon-Mueller, PhD, Anglia Ruskin University, Chelmsford, UK

Dr Alison B Rushton, EdD, Western University, Ontario, Canada

**Introduction**

Femoroacetabular Impingement syndrome (FAIS) is a well-recognised pathological entity and a common cause of hip and groin pain in young adults [1]. A recent epidemiological study reported a high prevalence of FAIS (47%), within a sample (n=200, aged 16-65 years) representative of the UK population [2].The pathology encompasses a morphological abnormality of the femur (Cam) or the acetabulum (Pincer) and can lead to chondro-labral dysfunction and eventually in some cases early onset of osteoarthritis [3].Additionally, deficits in muscle strength and range of motion (ROM) may lead to altered or increased hip joint loads causing detrimental effects [4, 5]. Non-surgical management such as physiotherapy will assist in modifying hip joint loads and address the above physical impairments, although, evidence of the effectiveness of such interventions are currently lacking [6].

Excision of the FAI lesion and addressing chondro-labral pathology through surgical procedures like hip arthroscopy may be necessary to provide better medium to long term outcomes [7]. Innovations in diagnosis and surgical techniques has resulted in an exponential growth of hip arthroscopy procedures in the past decade [8]. Two recent multicentre randomised controlled trials (RCTs) have found significant improvement in outcomes following hip arthroscopy compared to physiotherapy [9, 10]. However, results demonstrate that only half (51%) of participants showed significant improvement on the primary outcome measure - iHOT33 (mean difference 6·8 points; 95% CI 1·7–12·0) [9]. Several factors have been shown to influence the outcomes after hip arthroscopy. These include, the presence of osteoarthritis, severe dysplasia, inadequate removal of the impingement lesion, and soft tissue injury sustained during the procedure [3, 11, 12]. Amongst the non-surgical factors are prolonged waiting times leading to the chronicity and deconditioning of the muscles [13], deficits in muscle strength [14], psychological distress [15] and presence of concomitant pathologies like gluteal tendinopathy [16] and athletic pubalgia [17]. Therefore, early identification and optimisation prior to surgery will be crucial in delivering optimal post-operative outcomes.

Pre-operative rehabilitation intervention or ‘prehabilitation’ is the process of enhancing a patient’s functional capacity prior to surgery in order to improve post-operative outcomes [18]. Prehabilitation interventions have been tested and found to be beneficial across all age groups and various surgical pathways including orthopaedics [19, 20]. It is important for interventions included in the prehabilitation programme to be specific to the clinical condition, personalised and tailored to patient needs. A person-centred approach, which allows patients to take control of their own outcomes through prehabilitation, places the patient at the core of their peri-operative pathway [21]. This will heighten their motivation to make positive behavioural changes during the pre-operative phase and provide both peri and post-operative benefits [22].

Although the effectiveness of prehabilitation has been investigated in a wide variety of orthopaedic conditions, its effectiveness in patients undergoing hip arthroscopy has received little attention and not been studied in depth. Recently, a small pilot study on FAIS patients (n=18) favoured prehabilitation over standard care [23]. The findings suggested that a definitive trial to evaluate effectiveness would be valuable.

**Aim**

To evaluate the feasibility, suitability, acceptability and safety of a novel prehabilitation programme for FAIS to inform a future definitive randomised control trial to assess effectiveness.

**Objectives**

The objectives of the study and success criteria are detailed in Table 1.

**General Objectives**

To assess the feasibility, suitability, acceptability and safety of prehabilitation intervention including the following:

- Recruitment procedures
- Data collection methods
- Follow-up procedures
- Determine sample size for a full trial to test its effectiveness

**Specific objectives**

**Feasibility**

- To evaluate participant recruitment and attrition rates [24]
- To evaluate follow-up rates and response rates to questionnaires [24]
- To evaluate the ease of using the Physitrack app [25]
- To evaluate the time and effort of clinicians to deliver the intervention [24]
- To evaluate fidelity of intervention delivery [26]

**Suitability**

- To evaluate suitability of the outcome measures [27]
- To evaluate participant adherence with prehabilitation interventions [24]
- To evaluate the time taken to undertake each phases of the study [24]
- To evaluate the appropriateness of incorporating a novel prehabilitation intervention for FAIS into the current NHS services infrastructure [28]

**Acceptability**

- To evaluate the acceptability of interventions to patients and clinicians [27]
- To evaluate the willingness of patients to participate in a full RCT in the future [27]

**Safety**

- To evaluate the safety of the intervention [24]

**Methods**

This feasibility trial protocol is registered with ISRCTN Registry No. [15371248](https://gbr01.safelinks.protection.outlook.com/?url=https%3A%2F%2Furldefense.com%2Fv3%2F__https%3A%2Fgbr01.safelinks.protection.outlook.com%2F%3Furl%3Dhttps*3A*2F*2Fwww.isrctn.com*2FISRCTN15371248%26data%3D05*7C01*7Canuj.punnoose*40nhs.net*7C9fa1d1cb31ac4eea522208db208040f0*7C37c354b285b047f5b22207b48d774ee3*7C0*7C1*7C638139507488348179*7CUnknown*7CTWFpbGZsb3d8eyJWIjoiMC4wLjAwMDAiLCJQIjoiV2luMzIiLCJBTiI6Ik1haWwiLCJXVCI6Mn0*3D*7C3000*7C*7C*7C%26sdata%3DjhuBzsDF9dAXwZp2WybtzoFfhM2QmT*2BD5nBgph95ur8*3D%26reserved%3D0__%3BJSUlJSUlJSUlJSUlJSUlJSUlJSUl!!NLFGqXoFfo8MMQ!o5gyU8Yg-xtoOcV03CIwwOj7buBTB7wtM4htRAAEHbePs9xDAJES7cOV8pIh9cNPci9izQN619tq1p8G25HStGs06plu7McpEOMnDzY%24&data=05%7C01%7Canuj.punnoose%40nhs.net%7C7ea3de2b1fe449cc8fce08db208cf620%7C37c354b285b047f5b22207b48d774ee3%7C0%7C1%7C638139562080717319%7CUnknown%7CTWFpbGZsb3d8eyJWIjoiMC4wLjAwMDAiLCJQIjoiV2luMzIiLCJBTiI6Ik1haWwiLCJXVCI6Mn0%3D%7C3000%7C%7C%7C&sdata=qUiiP46We8crUy1mnwHx6QBKHD8yqnz7a3Iu3yI%2BYI4%3D&reserved=0) and reported as per  CONSORT 2010 statement: extension to randomised pilot and feasibility trials to ensure transparency and reproducibility [30]. All patient and public involvement meetings will be evaluated using an Impact log and reported in line with the GRIPP2 short form reporting checklist [31].

This feasibility trial will use a mixed methodology encompassing the following;

- A quantitative two arm parallel group feasibility trial
- Embedded qualitative component (Physiotherapists & Research participants) using thematic analysis

**Design**

A randomised control study design (RCT) is considered as gold standard when evaluating

the effectiveness of an intervention as it eliminates various biases prevalent in other study designs [32]. However, a RCT can be challenging with several unknown factors at play (recruitment, adherence, attrition, acceptability and suitability of interventions). Therefore, prior to evaluating the effectiveness of an intervention, researchers are encouraged to assess its feasibility, suitability of outcomes and optimise the overall design of the trial to ensure any uncertainties are addressed prior to a full trial [26, 33]. This study design will allow to test the feasibility of a novel prehabilitation intervention in an NHS setting, measure adherence, evaluate fidelity of intervention delivery and assess the suitability and acceptability of the interventions.

**Participant demographic data**

Participant baseline demographic data such as age, gender, height, weight, Body mass index, duration of symptoms and work status will be recorded. Additionally, details of their physical examination, investigations and past medical history recorded as a part of their routine care may be collected and used for the purpose of this study.

**Trial component**

A prospective two group parallel feasibility trial will be conducted to answer the above trial objectives [27].

Outcome measures will be obtained at baseline (prior to prehabilitation intervention), after prehabilitation before surgery, and at 6 weeks+/- 4 weeks and 6 months+/- 4 weeks (planned primary endpoint for definitive RCT) postoperatively when participants attend the research site for clinical care. Additionally, all patient reported outcome measures will be captured at 12 months+/-4 weeks post-op (secondary end point). Physical outcome measures will be taken onsite during the participant’s clinical visits and patient reported outcomes will be collected via the Physitrack app (<https://www.physitrack.com/clinical-studies>) or mailed to the participants at various follow-up points (see Fig. 2).

**Participants**

The study will be conducted at the Young Adult Hip Service in Addenbrooke’s – Cambridge University Hospital NHS Foundation Trust, Cambridge, UK, which has a good track record of recruiting to trials and managing them as well. Potential participants will be identified from the waiting list for hip arthroscopy at the NHS site. This will be performed by the Chief Investigator (CI) or the Principal Investigator (PI) who is a part of the direct clinical care team. Potential participants for the study will be contacted if their surgical date in the future will allow appropriate time for the consent process, randomisation and 8 weeks of prehabilitation intervention.

A Patient Information Sheet (PIS) will be provided in clinic or mailed to participants to facilitate the consenting process. The research team will ensure that they adequately explain the aim, study treatment, potential benefits or harm of taking part in the trial to the participants. They will also re-iterate that participation is voluntary and participants can withdraw from the study at any time, without giving a reason. Participants will be phoned after 1 week from when they have received the PIS to see if they are interested in taking part. For patients interested in participating, the consent process will be completed over the phone. The consent process will be witnessed by a member of staff independent of the trial team. The PI (or delegate) will sign the consent form as the person receiving consent and the independent witness will sign the consent form to confirm that the consent process was followed, and the participant gave their verbal consent to take part. Participants will be asked to sign the consent form when they come for their surgery, and they will receive a copy.

**Inclusion criteria**

All patients referred to Cambridge Young Adult Hip Service (aged 16-50 years), undergoing hip arthroscopy for the management of FAIS.

**Exclusion criteria:**

- Previous hip disease such as Perthe’s, Slipped upper femoral epiphysis or avascular necrosis [9]
- Participants who are unable to give full written consent
- Participants who are not fluent in English
- Pre-existing neuromuscular conditions like Motor Neuron Disease or Multiple Sclerosis
- History of any previous hip surgery
- History of any previous hip arthroscopies

**Randomisation and blinding**

Following baseline evaluation, participants will be randomly allocated to one of the two groups (prehabilitation or usual care) by the PI using a computer-generated random allocation sequence. This will be uploaded to a secure database known as REDCap (Research Electronic Data Capture) and all study personnel will be blinded to the allocation sequence ensuring adequate concealment [67, 68]. REDCap is a secure, web-based software platform designed to support data capture for research studies, providing 1) an intuitive interface for validated data capture; 2) audit trails for tracking data manipulation and export procedures; 3) automated export procedures for seamless data downloads to common statistical packages; and 4) procedures for data integration and interoperability with external sources.

Blinding participants and clinicians delivering interventions will not be possible as both will be aware of the intervention and allocation. However, the content of the programme will only be known to participants in the intervention group thereby reducing the risk of cross contamination between groups [34]. Physical outcome measures will be measured by a blinded assessor who will be trained by the PI to ensure standardisation and improve reliability of the assessment.

**Interventions**

All participants, regardless of group allocation, will receive standard peri & post-operative care. A record of their analgesia consumption will be noted during their follow-up appointments in a logbook by a member of the research team.

**Prehabilitation Intervention**

Interventions are described based on the Template for Intervention Description and Replication (TIDieR) [35]. The exercise intervention used in this study was developed utilising the findings of a systematic review on prehabilitation and an expert panel consensus methodology to define the core components of the intervention. The intervention will be delivered over a period of 8 weeks prior to surgery and will target 5 domains- muscle strength, range of motion, proprioception, cardiovascular fitness and addressing co-existing pathologies. Literature suggests high prevalence and poorer outcomes of depression and anxiety in people with FAIS [15, 36], and the intervention will therefore also include an in-person educational session delivered by the PI to provide greater understanding of the surgery to alleviate anxiety and help manage patient expectations. The intervention will include at least four in-clinic face-to-face sessions (once a fortnight) with an experienced physiotherapist (>2 years’ experience in treating musculoskeletal conditions including FAIS) followed by six to eight remotely monitored sessions (once a week) using a telehealth system. The number of sessions will be determined based on physiotherapist assessment and intensity and complexity of exercises will be gradually increased depending on each participant’s individual progress (Fig 1). Due to the wide variation of patient characteristics within the FAIS population (e.g. sedentary or athletic) interventions will be tailored according to individual participant’s capabilities.

**Use of Physitrack app and Telehealth system:**

Physitrack provides exercise information and videos via website and apps. Dosage and frequency of the exercises can be selected, and patients will be encouraged to record completion of the exercises enabling the physiotherapist to measure adherence via the app. Physitrack has been found to improve adherence and patient confidence and is utilised across several NHS Trusts in the UK [37]. Additionally, the use of Physitrack’s tele-rehabilitation would enable physiotherapists and patients to interact with each other via video providing reassurance of the correct exercise techniques and progression.

The participants in the intervention group will use Physitrack which provides photos and videos of the prescribed exercises and those unable to use the app will be provided with paper-based instructions. Additionally, patients will be asked to complete their exercises at home twice weekly.

**Training of Physiotherapists delivering the prehabilitation intervention**

The prehabilitation intervention will be delivered by experienced physiotherapists as described above. All the rehabilitation components are standard clinical therapies familiar to a trained physiotherapist. The physiotherapists will undergo training (by PI) on how to deliver these components together as per the protocol for this study. A representative from Physitrack will be invited to deliver training on the use of the app.

**Usual care intervention**

As per the host hospital’s current clinical guidelines, the usual care intervention consists of advice to continue with normal activities and no prehabilitation therapy.

**Qualitative component**

An embedded qualitative component will be utilised to answer specific trial objectives and to refine and adapt the design prior to the full RCT. Prior to participating in the qualitative study, participants will be provided with an information leaflet and opportunity will be given to raise any questions to the researchers regarding the processes. Consent from all participants will be obtained prior to commencement. All qualitative data will be recorded and transcribed verbatim. Transcripts will be returned to participants for review and edited prior to analysing the data.

**Physiotherapists**

In-depth face to face semi structured interviews will be used to evaluate the views of the Physiotherapists (n=4) regarding the feasibility, suitability and acceptability of the intervention, outcome measures as well as the Physitrack app in an NHS setting as described below. The interviews will be conducted by the PI within one year of commencement of the study to gather qualitative data. Appropriate questions for the interviews will be developed by the PI and the supervisory team. A patient and public involvement group will review the questions for clarity and appropriateness [38].

**Research participants**

Focus groups with research participants will be conducted following the 6 months assessment point to evaluate the research objectives. A purposive sample of 6-8 patients at different points of their research pathway (pre-op & post-op) will be included as recommended in the literature[39]. A predetermined topic guide developed by the PI and reviewed by the PPI panel will be used for the focus group. The focus group will be conducted by 2 researchers: the PI (facilitator) and a supervisor (observer) and two or more sessions maybe conducted until data saturation has been achieved.

**Outcome measures**

**Feasibility measures**

Feasibility parameters will be utilised to answer the objectives as per Table 1. The recruitment rate will be determined by the number of participants who are eligible and consent to participate in the study. Attrition will be defined by the number of consenting participants who drop out during the study. Timing of dropouts and reasons (where provided) will be explored to determine if the follow-up points are appropriate prior to a definitive RCT. Follow-up rates and response rates to questionnaires will be collected at each follow-up point. The usability of the Physitrack app and tele-rehabilitation will be evaluated using a modified System Usability Scale (SUS)[25]. Time and effort of clinicians delivering the interventions will be captured via the hospital’s electronic system.

Treatment fidelity is the extent to which an intervention is delivered as per study protocol and is critical in the development and testing of evidence-based interventions [40]. Assessing fidelity in the feasibility stage is also critical to identify interventions that have lower than expected fidelity which can then be used to refine interventions prior to a full trial [41]. Fidelity will be monitored by analysing self-reported checklists completed by the treating Physiotherapists. A numerical rating scale (5 point Likert scale) will be used to measure content and quality of the interventions and post-session feedback will be given to the clinicians to improve treatment delivery [42]^.^

**Suitability measures**

Physiotherapists and participants will be asked the appropriateness of outcome measures as well as time and effort required to complete them. Adherence to the intervention will be measured by capturing the number of exercises sessions delivered face-to-face and home exercises remotely monitored by Physitrack app. Qualitative data from the interviews and the focus group will also be collected to develop a training manual and to establish if there is adequate resources and infrastructure at the host hospital to support a definitive trial in the future.

**Acceptability measures**

Acceptability of the interventions will be evaluated during focus groups with participants using qualitative methods.

**Safety**

Safety of the interventions will be determined based on the number and nature of adverse events. All adverse events (related or unrelated to interventions) will be recorded. Components of the prehabilitation interventions are designed not to aggravate patient symptoms. However, in case of worsening of symptoms, the physiotherapist will re-evaluate and adapt the exercises to ensure safety.

**Primary clinical outcome measure**

**iHOT-12**

The iHOT-12 is a patient reported outcome of Quality of Life (QoL) [43] and will be the planned primary outcome measure for this study. This shorter version of the original iHOT-33 was developed and validated for patients with FAIS and was found to be valid, responsive and a reliable tool to measure the impact of hip disease in young active individuals on QoL [43].The minimal clinical important difference for the iHOT-12 is 13.0 and patient acceptable symptom state threshold is 63.0 for FAIS [44].

**Secondary clinical outcome measures**

**Muscle strength using Handheld dynamometer**

Several studies have discussed the implications of reduced muscle strength in the hip and how this could predispose to lower extremity injuries [45-47]. A recent systematic review evaluating muscle strength measurement in various hip pathologies reported that there is evidence of bilateral hip muscle weakness regardless of whether the hip joint is symptomatic or not [48]. Both motor driven and hand-held dynamometry (HHD) are reliable methods of muscle strength measurement and should be used with make tests (where the patient pushes against the examiner’s fixed resistance) [48]. Due to the pragmatic nature of this study HHD will be used. HHD with an external belt fixation has shown good inter-tester reliability with intra-class coefficient of 2.1 (range 0.76 to 0.95) [49]. Maximum voluntary isometric contraction will be utilised as the measurement for all hip muscles groups [50]. A minimum detectable change (MDC) score is often used to detect real change in an outcome measure overtime [51]. An MDC value at 95% CI ranging from 9-45 points was reported for individual hip muscle groups in healthy subjects using a HHD showing it is responsiveness [52]. Further details on how to perform the tests are described in supplementary Appendix 1.

**Star Excursion Balance Test (SEBT)**

The SEBT is a reliable measure to assess dynamic postural control and proprioception and has shown excellent inter-rater reliability [53]. The SEBT has shown good criterion and divergent validity in relation to pain, hip strength and ROM in patients with FAIS [54, 55]. See supplementary Appendix 1.

**Brief Pain Inventory-short form (BPI)**

Most patients with FAIS experience pain in the hip or groin [56], often chronic in nature. BPI is a multidimensional scale that can reliably measure chronic pain and its interference with an individual’s physical and social functioning [57]. The tool is responsive to change in pain associated with pharmacological, physical as well as psychological interventions and was recommended by the Initiative on Methods, Measurement, and Pain Assessment in Clinical Trials [58].

**Hospital Anxiety and Depression Scale (HADS)**

Recent studies have shown the prevalence of anxiety and depression in patients undergoing hip arthroscopic procedures [15, 36]. Previous studies have reported improvements in the level of anxiety and depression experienced by patients when undergoing prehabilitation [59, 60]. The HADS scale is a validated tool to measure anxiety and depression in general medical population of patients and will be utilised in the study [61]. A literature review including 747 papers reported a sensitivity and specificity of 0.8 at a cut off score of >8 on HADS which was very similar to other health questionnaires [62].

**Patient’s Global Impression of change (PGIC)**

The Global rating of change (GRC) scales are often used in research particularly within the musculoskeletal area[63]. Among the GRC scales, PGIC scale is frequently used to gather a patient’s perception of change after an intervention. The scale typically consists of 7 points which depicts a patient’s overall improvement from “very much improved” to “very much worse[64]. These scales have shown adequate reproducibility and sensitivity to change in a variety of disorders , and are easy to use and interpret[63].

**Data analysis**

Data will be analysed using SPSS version 28. Descriptive statistics, such as percentages (for rate calculations), means, standard deviations and mean change (if data are normally distributed) will be analysed to evaluate the distribution of scores. Standard deviations and effect sizes for outcome measure data will be used to calculate the required sample size for a definitive trial in the future.

All transcribed qualitative data will be thematically analysed [65]. The Personal assistant to the Chief investigator will be responsible for the transcriptions. As a member of staff, they will follow all the protocols of confidentiality and information governance procedures as per the host hospital's policy. Once used, all original recordings will be deleted. Transcripts will be reviewed by the participants prior to analysis by the researchers using Nvivo software[66]. For credibility and confirmability of the qualitative data, coding and themes will be analysed by the PI and primary academic supervisor (LCM).

**Data storage**

Study data will be collected and managed using REDCap electronic data capture tools hosted at the host institution. Additionally, personal data of participants will be stored securely on EDGE- a clinical research management platform approved by the host institution and will follow GDPR (General Data Protection Regulations) guidelines. All personal data used for the study will be destroyed at the end of the study. Anonymised quotations from the respondents might be used for dissemination such as peer reviewed publications or conferences. No single patient data will be reported or published ensuring confidentiality.

**Sample Size**

An overall participant sample of 50-60 (25-30 per arm) is recommended to estimate standard deviations for calculating sample size required for a full trial [69]. As a tertiary referral centre for hip arthroscopies, 350-375 new patients are referred to the service every year out of which an estimated 120 hip arthroscopies are performed/year.

**End of the study**

The end of the study will be declared when the last participant in the study has completed their 1-year post-op follow-up.

# **Discussion**

Despite the exponential growth and development of innovative surgical interventions for FAIS, it is estimated that nearly half (49%) receiving hip arthroscopy do not achieve optimal outcomes [9]. Deficits in muscle strength and ROM in patients with FAI is well evidenced in the literature [14, 70]. Additionally, pandemic outbreaks like COVID-19 has placed an unprecedented delay on elective operations leading to further chronicity and deconditioning in patients [71]. Therefore, optimising patients through effective prehabilitation programmes might play a vital role in early identification of impairments and promote physical and psychological wellbeing prior to undergoing surgery [72]. However, the role of exercise interventions prior to surgery in FAIS is under explored. This study will provide important preliminary data to inform feasibility of a definitive RCT in the future to evaluate effectiveness of a novel prehabilitation intervention. Additionally, the study will record compliance, adverse effects and satisfaction of the prehabilitation programme which are important elements at the feasibility stage [24].

It is anticipated that this programme will eventually lead to improvement in the delivery of FAIS care, reduce social and healthcare costs and assist in targeting and optimising patients much earlier in their clinical pathway.

**Ethics and dissemination**

This study raises no major ethical or legal issues.

The randomised study design will provide equal opportunities for all participants to treatment allocation. Each participant in the study will be given an opportunity to read and understand the study prior to signing the consent form and they will have the right to withdraw from the study at any time. The intervention will be delivered as a combination

of face-to-face and remotely using an app giving equal opportunities for patients who are unable to travel often. Reimbursements for their travel has been included and approved by the funding organisation.

Additionally, participation in the study will not alter their clinical pathway. Outcome measures will be taken at the time of their normal clinical visits reducing their visits to the hospital.

**Patient and Public Involvement (PPI)**

A focus group involving a patient and public panel informed the design of the study. An advisory group will be formed with 2 people from the PPI panel who will oversee the study. Several activities are planned with the PPI panel during the study as below;

1. Co-design the prehabilitation leaflet
2. Develop question guides for the research focus groups and semi structured interviews
3. Co-develop a training manual for clinicians
4. Design dissemination materials

**Strengths of the study**

- Interventions developed using a robust methodology (systematic review with meta-analysis and expert panel consensus)
- The first study to test the efficacy of a novel prehabilitation intervention in the FAIS group
- Randomised design and assessor blinding minimising selection, detection and performance biases.

**Limitations of the study**

- The number of study participants has been selected to test efficacy and feasibility to be able to perform a sample size calculation to determine the number of participants required for a definitive trial. Therefore, these results should be considered as preliminary findings.
- Study is conducted in a single tertiary hospital and therefore may need to be adapted prior to wider implementation.

**Impact of the study**

As this is a feasibility study, the impact of this study on patient care and NHS costs cannot be understood fully and may not change practice. However, if found successful further funding will be sought out for a future definite trial to test its effectiveness. A well-designed prehabilitation study will positively impact patient outcomes by improving strength and function and reducing anxiety prior to surgery thereby ensuring quicker recovery post-op. It is anticipated to reduce socio-economic burden by facilitating continuation or resumption of work and may reduce economic impact on services post-op (e.g., reduced need for physiotherapy follow-up and quicker discharge from the service). This study is conducted within a tertiary NHS setting and therefore can be reproduced and applied in similar healthcare settings.

**Funding**

This programme of research is conducted as part fulfilment of a PhD fellowship funded by the National institute for Health and Care Research, UK and hosted by Anglia Ruskin University, Cambridge, UK

**Competing interests**

None to declare

**Acknowledgements**

The authors would like to thank Physitrack PLC for permitting the use the images.

**Supporting information**

**Fig 1. Progression of Abductor strengthening exercise**

*Reprinted from****Physitrack PLC (***[***https://www.physitrack.com/***](https://www.physitrack.com/)*) under a CC BY license, with permission from****Physitrack PLC****, original copyright****2012***

Exercise

Progression


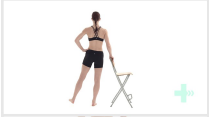


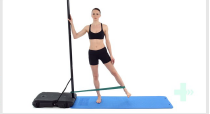


**Table 1. Study objectives and success criteria**

| General objectives | Success criteria |
| --- | --- |
| Recruitment procedure | Participants were recruited within the time constraints of the local NHS hospital |
|  | Participants report that there were no challenges with the recruitment procedure |
| Data collection methods | Data completeness of ≥80% |
|  | Patients and assessors reported that there were no challenges with the data collection methods |
| Follow-up procedures | 100% of participants were contacted for follow-up |
|  | ≥80% completion of follow-up outcome measures |
| Specific objectives | **Success criteria** |
| *Feasibility* | |
| Participant recruitment rates | The recruitment rate of this study will be considered sufficient if at least 33% of eligible patients are recruited over a period of 18 months (n=60). |
| Attrition rate | <30% dropout |
| Usability of Physitrack | Patients and Physiotherapists reported no challenges with the use of Physitrack and telehealth system. |
| Capacity (time and effort) of clinicians to deliver the programme | Physiotherapists report that they had adequate time and resources available to deliver the programme |
| Treatment fidelity | ≥80%  completion of the self-reported checklist |
|  | Physiotherapists reports that there were no barriers identified in the use of self-reported checklists. |
| *Suitability* | |
| Outcome measures | Patients and assessor report that the outcome measures were appropriate and self-explanatory |
|  | Data completeness of  ≥80% |
| Adherence to the programme | ≥70%  of the sessions (face to face + home ex monitored by Physitrack) |
| Time required to undertake each stages of the study | Physiotherapists report that they had enough time to complete each stages of the study. |
| Service infrastructure | Recruitment targets met  Data completeness of ≥80%  Clinicians and researchers report that there was adequate infrastructure to allow completion of a full trial. |
| *Acceptability* | |
| Intervention | Patients and clinicians report that the programme was appropriate and satisfactory |
| Randomisation | Patients are willing to be randomised for a future definitive trial.  (≥80% ) |
| Safety | |
| Intervention | Patients report that the intervention was safe, and no serious adverse events occurred during the prehabilitation phase. |

**Fig. 2. Timeline for interventions and outcome measures**

**Outcome measures**

- Feasibility outcomes
- iHot-12
- Muscle strength
- SEBT
- HADS
- Pain using BPI
- PGIC

**8 weeks intervention**

- 1 supervised group education
- 4 supervised in clinic sessions
- Up to 8 remotely supervised sessions using apps & Telehealth

Surgery

**Intervention period**

**Home assessment**

Patient reported outcomes

**In clinic assessment**

Feasibility measures

Patient reported outcomes

Physical outcomes

**In clinic assessment**

Feasibility measures

Patient reported outcomes

Physical outcomes

**Supplementary data**

**Interventions:**

The prehabilitation programme is based on the Madrid consensus statement as explained earlier and its delivery described using TIDieR checklist. The six key components targeted will include;

1. Muscle strength
2. Range of motion
3. Proprioception
4. Cardiovascular fitness
5. Address co-existing pathologies (e.g. gluteal, adductor tendinopathy) if any
6. Patient education to alleviate anxiety and better prepare for surgery

**Prehabilitation Phase 1 (0-4 weeks)**

This phase consists of 1 educational session (45 min) in a group setting, 2 fortnightly one to one session with a qualified Physiotherapist and 1 remotely supervised session weekly using Telehealth. Additionally, participants will be asked to carry on with an unsupervised home exercise programme twice weekly. Adherence will be monitored using Physitrack app.

**Prehabilitation Phase 2 (5-8 weeks)**

This phase consists 2 fortnightly one to one session with a qualified Physiotherapist and 1 remotely supervised session weekly using Telehealth. Additionally, participants will be asked to carry on with an unsupervised home exercise programme twice weekly. Adherence will be monitored using Physitrack app.

**Appendix 1**

**Hip muscle strength tests[6, 49]**

All hip strength tests (Isometric) will be done using a Handheld dynamometer (HHD). Each strength test will be performed 3 times- 2 reps with submaximal force and then the 3^rd^ attempt as hard as possible and hold for 5 seconds. Rest time of 5 seconds will be allowed between each repetitions and a 30 seconds minimum between each test. An external belt will be used to fix the HHD to improve inter-rater reliability.[49]

**Supine position**

***Abduction strength[52]***

Test leg resting in neutral

Participant will be asked to hold onto the exam table to stabilise the trunk

HHD will be placed 5 cm above the lateral malleolus of the testing limb and fixed with an external belt attached to a handle fixed onto the wall.

Instruction: ‘ Go ahead, push, push, push and relax’.

***Adduction strength[52]***

Test leg resting in neutral

Participant will be asked to hold onto the exam table to stabilise the trunk

HHD will be placed 5 cm above the medial malleolus of the testing limb and fixed with an external belt attached to a handle fixed onto the wall.

Instruction: ‘ Go ahead, push, push, push and relax’.

**Prone position**

***Extension strength[6]***

Test leg knee bend at 90 deg and placed on the edge of the foot of the exam table

Participant will be asked to hold onto the exam table to stabilise the trunk

HHD will be placed over the centre of the heel of the testing limb and fixed with an external belt attached to a handle fixed on the floor.

Instruction: ‘Go ahead, push the foot towards the ceiling, push, push, push and relax’.

**Sitting**

***Flexion strength[52]***

Participant sitting on the edge of the exam table with knee flexed at 90 deg

Participant will be asked to hold onto the exam table with both hands to stabilise the body

HHD placed 5cm above the superior pole of the patella and fixed with an external belt attached to a handle fixed on the floor.

Instruction: ‘Go ahead, pull your knee up to the ceiling, pull, pull, pull and relax’

***Internal rotation strength[52]***

Participant sitting on the edge of the exam table with both knees flexed at 90 deg

Participant will be asked to hold onto the exam table with both hands to stabilise the body

HHD placed 5cm above the lateral malleolus fixed with an external belt attached to a handle fixed on the floor.

Instruction: ‘Go ahead, push, push, push and relax’

***External rotation strength[52]***

Participant sitting on the edge of the exam table with both knees flexed at 90 deg

Participant will be asked to hold onto the exam table with both hands to stabilise the body

HHD placed 5cm above the medial malleolus and fixed with an external belt attached to a handle fixed on the floor.

Instruction: ‘Go ahead, push, push, push and relax’

**Side lying**

***Trunk endurance[73]***

Participant will be in the side lying position with the hip resting on the exam table or the floor mat and one leg resting over the other.

Participant will be instructed to lift the hip off the floor and hold the position for as long as possible supporting the weight through the forearm and feet.

The time (seconds) will be recorded from the start until the end of the test when the participant’s hip touches the floor.

Instruction: ‘Go on, lift your hip off the floor and hold the position as much as possible’.

Verbal prompts will be given to the patient every 30 sec.

**Functional Test**

**Star Excursion Balance Test (SEBT)[6, 54]**

Anterior

Anteromedial

Anterolateral

Lateral

Medial

Posterolateral

Posteromedial

Posterior

**Fig 1. SEBT directions for left leg stance**

We will measure 4 different directions namely anterior, anteromedial, posteromedial and posterolateral.

The participant will be asked to stand on one leg at the centre of the circle. While maintaining the stance, they will be asked to reach with the other foot as far as possible and touch the line with their big toe. The test will be performed starting from the anterior direction clockwise. The assessor will measure the distance in all four directions. The test will be invalid if the participant (i) failed to maintain stance, (ii) lifts or moves the stance foot from the centre point, (iii) touches the reach foot down fully and (iv) Unable to bring the reach foot back to the starting position.

The participant will be allowed a trial attempt on either legs and test will be conducted on both sides.

**References**

1. Griffin DR, Dickenson EJ, O'Donnell J, Agricola R, Awan T, Beck M, et al. The Warwick Agreement on femoroacetabular impingement syndrome (FAI syndrome): an international consensus statement. Br J Sports Med. 2016;50(19):1169-76.

2. Dickenson EJ, Wall PDH, Hutchinson CE, Griffin DR. The prevalence of cam hip morphology in a general population sample. Osteoarthritis Cartilage. 2019;27(3):444-8.

3. Viswanath A, Khanduja V. Can hip arthroscopy in the presence of arthritis delay the need for hip arthroplasty? J Hip Preserv Surg. 2017;4(1):3-8.

4. Lewis CL, Sahrmann SA, Moran DW. Anterior hip joint force increases with hip extension, decreased gluteal force, or decreased iliopsoas force. J Biomech. 2007;40(16):3725-31.

5. Alshameeri Z, Khanduja V. The effect of femoro-acetabular impingement on the kinematics and kinetics of the hip joint. Int Orthop. 2014;38(8):1615-20.

6. Kemp JL, Johnston RTR, Coburn SL, Jones DM, Schache AG, Mentiplay BF, et al. Physiotherapist-led treatment for femoroacetabular impingement syndrome (the PhysioFIRST study): a protocol for a participant and assessor-blinded randomised controlled trial. BMJ Open. 2021;11(4):e041742.

7. Holleyman R, Sohatee MA, Lyman S, Malviya A, Khanduja V, Group NU. Hip arthroscopy for femoroacetabular impingement is associated with significant improvement in early patient reported outcomes: analysis of 4963 cases from the UK non-arthroplasty registry (NAHR) dataset. Knee Surg Sports Traumatol Arthrosc. 2022.

8. Palmer AJ, Malak TT, Broomfield J, Holton J, Majkowski L, Thomas GE, et al. Past and projected temporal trends in arthroscopic hip surgery in England between 2002 and 2013. BMJ Open Sport Exerc Med. 2016;2(1):e000082.

9. Griffin DR, Dickenson EJ, Wall PDH, Achana F, Donovan JL, Griffin J, et al. Hip arthroscopy versus best conservative care for the treatment of femoroacetabular impingement syndrome (UK FASHIoN): a multicentre randomised controlled trial. Lancet. 2018;391(10136):2225-35.

10. Palmer AJR, Ayyar Gupta V, Fernquest S, Rombach I, Dutton SJ, Mansour R, et al. Arthroscopic hip surgery compared with physiotherapy and activity modification for the treatment of symptomatic femoroacetabular impingement: multicentre randomised controlled trial. BMJ. 2019;364:l185.

11. Shin JJ, de Sa DL, Burnham JM, Mauro CS. Refractory pain following hip arthroscopy: evaluation and management. J Hip Preserv Surg. 2018;5(1):3-14.

12. Kuroda Y, Saito M, Cinar EN, Norrish A, Khanduja V. Patient-related risk factors associated with less favourable outcomes following hip arthroscopy. Bone Joint J. 2020;102-B(7):822-31.

13. Basques BA, Waterman BR, Ukwuani G, Beck EC, Neal WH, Friel NA, et al. Preoperative Symptom Duration Is Associated With Outcomes After Hip Arthroscopy. Am J Sports Med. 2019;47(1):131-7.

14. Freke MD, Kemp J, Svege I, Risberg MA, Semciw A, Crossley KM. Physical impairments in symptomatic femoroacetabular impingement: a systematic review of the evidence. Br J Sports Med. 2016;50(19):1180.

15. Martin RL, Christoforetti JJ, McGovern R, Kivlan BR, Wolff AB, Nho SJ, et al. The Impact of Depression on Patient Outcomes in Hip Arthroscopic Surgery. Orthop J Sports Med. 2018;6(11):2325967118806490.

16. Speers CJ, Bhogal GS. Greater trochanteric pain syndrome: a review of diagnosis and management in general practice. Br J Gen Pract. 2017;67(663):479-80.

17. Hammoud S, Bedi A, Magennis E, Meyers WC, Kelly BT. High incidence of athletic pubalgia symptoms in professional athletes with symptomatic femoroacetabular impingement. Arthroscopy. 2012;28(10):1388-95.

18. Ditmyer MM, Topp R, Pifer M. Prehabilitation in preparation for orthopaedic surgery. Orthop Nurs. 2002;21(5):43-51; quiz 2-4.

19. Moyer R, Ikert K, Long K, Marsh J. The Value of Preoperative Exercise and Education for Patients Undergoing Total Hip and Knee Arthroplasty: A Systematic Review and Meta-Analysis. JBJS Rev. 2017;5(12):e2.

20. Shaarani SR, O'Hare C, Quinn A, Moyna N, Moran R, O'Byrne JM. Effect of prehabilitation on the outcome of anterior cruciate ligament reconstruction. Am J Sports Med. 2013;41(9):2117-27.

21. Durrand J, Singh SJ, Danjoux G. Prehabilitation. Clin Med (Lond). 2019;19(6):458-64.

22. McDonald S, Yates D, Durrand JW, Kothmann E, Sniehotta FF, Habgood A, et al. Exploring patient attitudes to behaviour change before surgery to reduce peri-operative risk: preferences for short- vs. long-term behaviour change. Anaesthesia. 2019;74(12):1580-8.

23. Grant LF, Cooper DJ, Conroy JL. The HAPI 'Hip Arthroscopy Pre-habilitation Intervention' study: does pre-habilitation affect outcomes in patients undergoing hip arthroscopy for femoro-acetabular impingement? J Hip Preserv Surg. 2017;4(1):85-92.

24. Tickle-Degnen L. Nuts and bolts of conducting feasibility studies. Am J Occup Ther. 2013;67(2):171-6.

25. Bangor A, Kortum PT, Miller JT. An Empirical Evaluation of the System Usability Scale. International Journal of Human–Computer Interaction. 2008;24(6):574-94.

26. El-Kotob R, Giangregorio LM. Pilot and feasibility studies in exercise, physical activity, or rehabilitation research. Pilot Feasibility Stud. 2018;4:137.

27. Arain M, Campbell MJ, Cooper CL, Lancaster GA. What is a pilot or feasibility study? A review of current practice and editorial policy. BMC Med Res Methodol. 2010;10:67.

28. Bowen DJ, Kreuter M, Spring B, Cofta-Woerpel L, Linnan L, Weiner D, et al. How we design feasibility studies. Am J Prev Med. 2009;36(5):452-7.

29. Singal AG, Higgins PD, Waljee AK. A primer on effectiveness and efficacy trials. Clin Transl Gastroenterol. 2014;5:e45.

30. Eldridge SM, Chan CL, Campbell MJ, Bond CM, Hopewell S, Thabane L, et al. CONSORT 2010 statement: extension to randomised pilot and feasibility trials. Pilot Feasibility Stud. 2016;2:64.

31. Staniszewska S, Brett J, Simera I, Seers K, Mockford C, Goodlad S, et al. GRIPP2 reporting checklists: tools to improve reporting of patient and public involvement in research. Res Involv Engagem. 2017;3:13.

32. Hariton E, Locascio JJ. Randomised controlled trials - the gold standard for effectiveness research: Study design: randomised controlled trials. BJOG. 2018;125(13):1716.

33. Craig P, Dieppe P, Macintyre S, Michie S, Nazareth I, Petticrew M. Developing and evaluating complex interventions: the new Medical Research Council guidance. Int J Nurs Stud. 2013;50(5):587-92.

34. Sussman JB, Hayward RA. An IV for the RCT: using instrumental variables to adjust for treatment contamination in randomised controlled trials. BMJ. 2010;340:c2073.

35. Hoffmann TC, Glasziou PP, Boutron I, Milne R, Perera R, Moher D, et al. Better reporting of interventions: template for intervention description and replication (TIDieR) checklist and guide. BMJ. 2014;348:g1687.

36. Sochacki KR, Brown L, Cenkus K, Di Stasi S, Harris JD, Ellis TJ. Preoperative Depression Is Negatively Associated With Function and Predicts Poorer Outcomes After Hip Arthroscopy for Femoroacetabular Impingement. Arthroscopy. 2018;34(8):2368-74.

37. Bennell KL, Marshall CJ, Dobson F, Kasza J, Lonsdale C, Hinman RS. Does a Web-Based Exercise Programming System Improve Home Exercise Adherence for People With Musculoskeletal Conditions?: A Randomized Controlled Trial. American Journal of Physical Medicine & Rehabilitation. 2019;98(10).

38. Brett J, Staniszewska S, Mockford C, Herron-Marx S, Hughes J, Tysall C, et al. Mapping the impact of patient and public involvement on health and social care research: a systematic review. Health Expect. 2014;17(5):637-50.

39. Bloor M. Focus groups in social research: Sage; 2001.

40. Hildebrand MW, Host HH, Binder EF, Carpenter B, Freedland KE, Morrow-Howell N, et al. Measuring treatment fidelity in a rehabilitation intervention study. Am J Phys Med Rehabil. 2012;91(8):715-24.

41. Toomey E, Hardeman W, Hankonen N, Byrne M, McSharry J, Matvienko-Sikar K, et al. Focusing on fidelity: narrative review and recommendations for improving intervention fidelity within trials of health behaviour change interventions. Health Psychology and Behavioral Medicine. 2020;8(1):132-51.

42. Borrelli B. The assessment, monitoring, and enhancement of treatment fidelity in public health clinical trials. J Public Health Dent. 2011;71 Suppl 1:S52-63.

43. Griffin DR, Parsons N, Mohtadi NG, Safran MR, Multicenter Arthroscopy of the Hip Outcomes Research N. A short version of the International Hip Outcome Tool (iHOT-12) for use in routine clinical practice. Arthroscopy. 2012;28(5):611-6; quiz 6-8.

44. Nwachukwu BU, Chang B, Beck EC, Neal WH, Movassaghi K, Ranawat AS, et al. How Should We Define Clinically Significant Outcome Improvement on the iHOT-12? HSS J. 2019;15(2):103-8.

45. Khayambashi K, Ghoddosi N, Straub RK, Powers CM. Hip Muscle Strength Predicts Noncontact Anterior Cruciate Ligament Injury in Male and Female Athletes: A Prospective Study. Am J Sports Med. 2016;44(2):355-61.

46. Powers CM, Ghoddosi N, Straub RK, Khayambashi K. Hip Strength as a Predictor of Ankle Sprains in Male Soccer Players: A Prospective Study. J Athl Train. 2017;52(11):1048-55.

47. Mucha MD, Caldwell W, Schlueter EL, Walters C, Hassen A. Hip abductor strength and lower extremity running related injury in distance runners: A systematic review. J Sci Med Sport. 2017;20(4):349-55.

48. Mayne E, Memarzadeh A, Raut P, Arora A, Khanduja V. Measuring hip muscle strength in patients with femoroacetabular impingement and other hip pathologies: A systematic review. Bone Joint Res. 2017;6(1):66-72.

49. Thorborg K, Bandholm T, Holmich P. Hip- and knee-strength assessments using a hand-held dynamometer with external belt-fixation are inter-tester reliable. Knee Surg Sports Traumatol Arthrosc. 2013;21(3):550-5.

50. Meldrum D, Cahalane E, Conroy R, Fitzgerald D, Hardiman O. Maximum voluntary isometric contraction: reference values and clinical application. Amyotroph Lateral Scler. 2007;8(1):47-55.

51. de Vet HC, Terwee CB, Ostelo RW, Beckerman H, Knol DL, Bouter LM. Minimal changes in health status questionnaires: distinction between minimally detectable change and minimally important change. Health Qual Life Outcomes. 2006;4:54.

52. Thorborg K, Petersen J, Magnusson SP, Holmich P. Clinical assessment of hip strength using a hand-held dynamometer is reliable. Scand J Med Sci Sports. 2010;20(3):493-501.

53. Gribble PA, Kelly SE, Refshauge KM, Hiller CE. Interrater reliability of the star excursion balance test. J Athl Train. 2013;48(5):621-6.

54. Johansson AC, Karlsson H. The Star Excursion Balance Test: Criterion and divergent validity on patients with femoral acetabular impingement. Man Ther. 2016;26:104-9.

55. Freke M, Kemp J, Semciw A, Sims K, Russell T, Singh P, et al. Hip Strength and Range of Movement Are Associated With Dynamic Postural Control Performance in Individuals Scheduled for Arthroscopic Hip Surgery. J Orthop Sports Phys Ther. 2018;48(4):280-8.

56. Clohisy JC, Knaus ER, Hunt DM, Lesher JM, Harris-Hayes M, Prather H. Clinical presentation of patients with symptomatic anterior hip impingement. Clin Orthop Relat Res. 2009;467(3):638-44.

57. Cleeland CS, Ryan KM. Pain assessment: global use of the Brief Pain Inventory. Ann Acad Med Singap. 1994;23(2):129-38.

58. Turk DC, Dworkin RH, McDermott MP, Bellamy N, Burke LB, Chandler JM, et al. Analyzing multiple endpoints in clinical trials of pain treatments: IMMPACT recommendations. Initiative on Methods, Measurement, and Pain Assessment in Clinical Trials. Pain. 2008;139(3):485-93.

59. Lindback Y, Tropp H, Enthoven P, Abbott A, Oberg B. PREPARE: Pre-surgery physiotherapy for patients with degenerative lumbar spine disorder: a randomized controlled trial protocol. BMC Musculoskelet Disord. 2016;17:270.

60. Parker PA, Pettaway CA, Babaian RJ, Pisters LL, Miles B, Fortier A, et al. The effects of a presurgical stress management intervention for men with prostate cancer undergoing radical prostatectomy. J Clin Oncol. 2009;27(19):3169-76.

61. Zigmond AS, Snaith RP. The hospital anxiety and depression scale. Acta Psychiatr Scand. 1983;67(6):361-70.

62. Bjelland I, Dahl AA, Haug TT, Neckelmann D. The validity of the Hospital Anxiety and Depression Scale. An updated literature review. J Psychosom Res. 2002;52(2):69-77.

63. Kamper S. Global Rating of Change scales. Aust J Physiother. 2009;55(4):289.

64. Dworkin RH, Turk DC, Wyrwich KW, Beaton D, Cleeland CS, Farrar JT, et al. Interpreting the clinical importance of treatment outcomes in chronic pain clinical trials: IMMPACT recommendations. J Pain. 2008;9(2):105-21.

65. Braun V, Clarke V. Using thematic analysis in psychology. Qualitative Research in Psychology. 2006;3:77-101.

66. Welsh E. Dealing with Data: Using NVivo in the Qualitative Data Analysis Process. Forum: Qualitative Social Research. 2002;3.

67. Harris PA, Taylor R, Thielke R, Payne J, Gonzalez N, Conde JG. Research electronic data capture (REDCap)--a metadata-driven methodology and workflow process for providing translational research informatics support. J Biomed Inform. 2009;42(2):377-81.

68. Harris PA, Taylor R, Minor BL, Elliott V, Fernandez M, O'Neal L, et al. The REDCap consortium: Building an international community of software platform partners. J Biomed Inform. 2019;95:103208.

69. Sim J, Lewis M. The size of a pilot study for a clinical trial should be calculated in relation to considerations of precision and efficiency. J Clin Epidemiol. 2012;65(3):301-8.

70. Diamond LE, Dobson FL, Bennell KL, Wrigley TV, Hodges PW, Hinman RS. Physical impairments and activity limitations in people with femoroacetabular impingement: a systematic review. Br J Sports Med. 2015;49(4):230-42.

71. Uimonen M, Kuitunen I, Paloneva J, Launonen AP, Ponkilainen V, Mattila VM. The impact of the COVID-19 pandemic on waiting times for elective surgery patients: A multicenter study. PLoS One. 2021;16(7):e0253875.

72. Silver JK. Prehabilitation May Help Mitigate an Increase in COVID-19 Peripandemic Surgical Morbidity and Mortality. (1537-7385 (Electronic)).

73. Kemp JL, Risberg MA, Schache AG, Makdissi M, Pritchard MG, Crossley KM. Patients With Chondrolabral Pathology Have Bilateral Functional Impairments 12 to 24 Months After Unilateral Hip Arthroscopy: A Cross-sectional Study. J Orthop Sports Phys Ther. 2016;46(11):947-56.
