# Supplementary material for: PREHAB FAI- Prehabilitation for patients undergoing arthroscopic hip surgery for Femoroacetabular Impingement Syndrome -Protocol for an assessor blinded randomised controlled feasibility study
Source: PLoS One. 2024 Apr 11;19(4):e0301194. doi: 10.1371/journal.pone.0301194 (PMC11008823; doi:10.1371/journal.pone.0301194)
Supplement: Attachment — Submitted filename: PLOS one Rebuttal.docx [file pone.0301194.s006.docx]

| Reviewer comments | Author responses |
| --- | --- |
| **Editor** | |
| 1. Please ensure that your manuscript meets PLOS ONE's style requirements, including those for file naming. | Reviewed the guidelines and made the necessary changes as per recommendations |
| 2. We note that the grant information you provided in the ‘Funding Information’ and ‘Financial Disclosure’ sections do not match. | This has been verified and corrected |
| 3. Thank you for stating the following in the Competing Interests section:  "I have read the journal's policy and the authors of this manuscript have the following competing interests: Dr Khanduja reported being a chief investigator for the NIHR doctoral fellowship during the conduct of the study; receiving consulting fees from Smith & Nephew PLC and Arthrex Inc, outside the submitted work; serving as a board member for the UK Non-Arthroplasty Hip Registry, British Hip Society, European Society of Sports Traumatology, Knee Surgery, and Arthroscopy, and the International Society of Orthopaedic Surgery and Traumatology; and serving on the editorial boards of the Journal of Clinical Orthopaedics and Trauma, International Journal of Orthopaedics, International Orthopaedics, and Open Access Journal of Sports Medicine. No other disclosures were reported."   Please confirm that this does not alter your adherence to all PLOS ONE policies on sharing data and materials, by including the following statement: "This does not alter our adherence to PLOS ONE policies on sharing data and materials.” (as detailed online in our guide for authors http://journals.plos.org/plosone/s/competing-interests).  If there are restrictions on sharing of data and/or materials, please state these. Please note that we cannot proceed with consideration of your article until this information has been declared.  Please include your updated Competing Interests statement in your cover letter; we will change the online submission form on your behalf. | This has been added to the covering letter |
| 4. In your Data Availability statement, you have not specified where the minimal data set underlying the results described in your manuscript can be found. PLOS defines a study's minimal data set as the underlying data used to reach the conclusions drawn in the manuscript and any additional data required to replicate the reported study findings in their entirety. All PLOS journals require that the minimal data set be made fully available. For more information about our data policy, please see <http://journals.plos.org/plosone/s/data-availability>.   "Upon re-submitting your revised manuscript, please upload your study’s minimal underlying data set as either Supporting Information files or to a stable, public repository and include the relevant URLs, DOIs, or accession numbers within your revised cover letter | A data availability statement has been added as follows  “Deidentified research data can be made available upon request when the study is completed and published” |
| 5. PLOS requires an ORCID iD for the corresponding author in Editorial Manager on papers submitted after December 6th, 2016. Please ensure that you have an ORCID iD and that it is validated in Editorial Manager. | This has been linked |
| 6. Your ethics statement should only appear in the Methods section of your manuscript. If your ethics statement is written in any section besides the Methods, please move it to the Methods section and delete it from any other section. | This has been moved to Methods as recommended. |
| 7. We note that Figure 2 in your submission contain copyrighted images. All PLOS content is published under the Creative Commons Attribution License (CC BY 4.0), which means that the manuscript, images, and Supporting Information files will be freely available online, and any third party is permitted to access, download, copy, distribute, and use these materials in any way, even commercially, with proper attribution   Please upload the completed Content Permission Form or other proof of granted permissions as an "Other" file with your submission.    In the figure caption of the copyrighted figure, please include the following text: “Reprinted from [ref] under a CC BY license, with permission from [name of publisher], original copyright [original copyright year].” | Figure 2 has been removed.  However, the completed content permission form has been uploaded and caption has been added as recommended at the bottom of the S1 file for all other images from Physitrack PLC |
| 8. Please include captions for your Supporting Information files at the end of your manuscript, and update any in-text citations to match accordingly. Please see our Supporting Information guidelines for more information: http://journals.plos.org/plosone/s/supporting-information. | This has been included at the end of the manuscript as recommended |
| 9. We note that S1 File includes an image of a [patient / participant / in the study]. As per the PLOS ONE policy (http://journals.plos.org/plosone/s/submission-guidelines#loc-human-subjects-research) on papers that include identifying, or potentially identifying, information, the individual(s) or parent(s)/guardian(s) must be informed of the terms of the PLOS open-access (CC-BY) license and provide specific permission for publication of these details under the terms of this license. | The S1 file image is from Physitrack PLC and the content permission form has been uploaded as mentioned above. |
| **Reviewer 1** |  |
| 1. 88 which outcomes showed significant improvement? And at what time point? | The sentence has been edited as follows:  Furthermore, two recent multicentre randomised controlled trials (RCTs) have found significant improvement in outcomes relating to daily activities and quality of life at 8-12 months following hip arthroscopy compared to physiotherapy |
| 2. Which patient group had improvements? (Surgery vs non surgery results are not clear here when randomised control trials have shown similar function at 24 months between groups) | The sentence has been edited as follows:  The mean between group difference in the iHOT-33 scores was 6·8; 95% CI 1·7–12·0, p=0.0093 in favour of hip arthroscopy which exceeded the minimum clinically important difference (MCID) of 6.1 [10]. However, results from these trials demonstrate that only half (51%) of participants in the hip arthroscopy group showed significant improvement on the primary outcome measure– iHOT33 which relates to quality of life at 12 months post-op [10]. Another study from the Non Arthroplasty Hip Registry in the UK of over 4900 patients with FAI, showed only 35% of all patients improve or achieve optimal outcome post-operatively [12]. |
| 3. Will participants be given the option to have non-surgical management if they see significant improvements with prehabilitation over the 8-week period from their baseline measurements? | A very valid point and we are hoping that this would be case. This is a feasibility study and plans for changing the pathway is not currently within the remit of this study. However, we will be capturing the data through the qualitative focus group with participants and perhaps implement this in a future definitive trial. |
| **Reviewer 2** |  |
| 10. Consider allocating participants before baseline evaluation to minimize bias and enhance the validity of results. | Agree fully and this was a mistake in the manuscript. This has been changed to:  A simple random allocation sequence will be generated using GraphPad QuickCalcs website [41] and uploaded to REDCap. Once patients are allocated to one of the two groups (prehabilitation or standard care), all baseline data will be obtained. All study personnel will be blinded to the allocation sequence ensuring adequate concealment [35, 36]. |
| 11. Abstract:   I suggest that the author mention what outcome will be measured in the method section for better understanding. | This has been added in the abstract as follows  ‘Patient reported outcomes such as iHOT-12, Brief Pain Inventory Scale (Short form), Hospital Anxiety and Depression Scale and Patient Global Impression of Change score will be obtained alongside objective measurements such as Muscle Strength and Star Excursion Balance Test at various time points. |
| 12. Page 4, lines 74-76 “An epidemiological study conducted in 2015 reported a high prevalence of FAI (47%, 95% CI 42,51), within a sample (n=200, aged 16-65 years) representative of the UK population”.   This sentence is a bit hard to follow, maybe try to reword the sentence and emphasize the high prevalence in the UK population with specific values, e.g. “An epidemiological study revealed a high prevalence of FAI, with 47% of the sample ….”.  Please notice the correct format of the confidence interval (95%CI: [42-51]). | The sentence has been changed to  An epidemiological study on 200 patients aged 16-65 years, representative of the UK population, reported a high prevalence of FAI with 47% (95% CI [42-51]) showing signs of cam morphology [2]. |
| 13. Page 4, lines 90-92 “However, results from these trials demonstrate that only half (51%) 91 of participants showed significant improvement on the primary outcome measure – iHOT33 (mean difference 6·8 points; 95% CI 1·7–12·0) [10]”.   Does ‘these trials’ represent both surgery and physiotherapy? The author mentioned iHOT33 to demonstrate the evidence, but you need to specify what this outcome measured for, i.e., quality of life. | The sentence has been changed to:  However, results from these trials demonstrate that only half (51%) of participants in the hip arthroscopy group showed significant improvement on the primary outcome measure– iHOT33 which relates to quality of life at 12 months post-op [10]. |
| 14. S1 File   The programme is based on the consensus statement as evidence-based practice, the author needs to make sure the reference is cited. | The consensus paper was presented at ISHA annual scientific meeting in Oct 2023 and the reference has been cited. |
| 15. Page 9, lines 187-191 “…after prehabilitation before surgery, and at 6 weeks+/- 4 weeks and 6 months+/- 4 weeks (planned primary endpoint for definitive RCT) postoperatively when participants attend the research site for clinical care. Additionally, all patient-reported outcome measures will be captured at 12 months+/-4 weeks post-op.’’   Are the programme phases designed according to the evidence of systematic review or consensus statement? It is better to bring some evidence to illustrate why this particular timeline (in the introduction section), to strengthen the purpose of this study. | Agree. The following paragraphs have been added under quantitative component:  It is imperative that steps are undertaken to improve the methodological rigour right at the initial stages of any research study [34]. Consistent with the Medical Research Council Framework recommendations for developing and evaluating complex interventions [33], it is crucial to develop the intervention prior to testing its efficacy or effectiveness. A recent systematic review and meta-analysis conducted by the lead authors Punnoose & Claydon-Mueller et al., analysed 48 unique trials investigating the effectiveness of prehabilitation in patients undergoing orthopaedic surgery [22]. This study included both published and unpublished trials until June 2023 and synthesised evidence on the effectiveness of prehabilitation on several outcome domains including quality of life, pain, muscle strength and function, laying a thorough foundation to the prehabilitation intervention used in this study. Alongside underscoring the effectiveness of prehabilitation, the study also reported on the optimum duration, dosage and frequency of prehabilitation, and emphasised on the importance of reporting adherence to the exercise intervention. However, majority of studies (85%, n=41) included in the systematic review were on joint replacements, highlighting the lack of good quality research around prehabilitation effectiveness in other orthopaedic surgical procedures such as Anterior cruciate ligament reconstruction and FAIS.  Due to the lacunae of literature around prehabilitation components and its parameters within the FAIS population, an expert panel consensus statement with 17 international experts defined the core components of the intervention. The study recommended 6 core domains to be targeted during the pre-operative phase namely, muscle strength, range of motion, proprioception, cardiovascular fitness, anxiety and depression and addressing any co-existing pathologies that are present alongside FAIS. Additionally, the consensus statement recommended that prehabilitation for FAIS be delivered for 6-8 weeks as a combination of face to face and virtually via telehealth. This study is currently in its preparatory stages of publication. The resulting prehabilitation intervention developed though the rigorous process is detailed in S1 file. |
| 16. Page 9, line 201: “Chief Investigator (CI)”   The author mentioned confidence interval as CI in the previous Introduction section, but the same abbreviation appears in the Method section. Distinguish between "CI" as Chief Investigator and "CI" as a confidence interval for clarity. | Agree. This has been changed to Chief Investigator in full. |
| 17. Page 16 Randomization and blinding   “Following baseline evaluation, participants will be randomly allocated to one of the two groups (prehabilitation or usual care) by the PI using a computer-generated simple random allocation sequence.” If participants are allocated after baseline evaluation, there's a risk that the group assignments could be influenced by the knowledge of individual participants' baseline characteristics, and this could introduce bias into the study. Therefore, I recommend the author consider allocating participants before any baseline evaluations or interventions applied. This approach helps maintain the validity and scientific evidence of the results. Otherwise, you need to justify why the allocation is after the baseline evaluation.  This section needs to make more efforts to improve the study’s transparency and clarity. It is better to rearrange the section's placement for better flow, i.e., put this section before the outcome measurements. | Agree. The sentence has been changed to  A simple random allocation sequence will be generated using GraphPad QuickCalcs website [41] and uploaded to REDCap. Once patients are allocated to one of the two groups (prehabilitation or standard care), all baseline data will be obtained. All study personnel will be blinded to the allocation sequence ensuring adequate concealment [35, 36].  Agree. The section has been moved as per the recommendation. |
| 18. Page 24, lines 543-549   The author needs to improve the description of the study's strengths, focusing on its potential impact on future research and benefits to patients. The author should consider what the outcome could bring to future research, what benefit could bring to the patients who need surgical intervention, and whether qualitative research could have an impact on the participants. | Agree. The paragraph has been edited as follows  To our knowledge, this is the first study to test the feasibility, suitability, acceptability and safety of a newly developed prehabilitation intervention in patients undergoing hip arthroscopy for FAIS. The prehabilitation intervention was developed using a robust methodology underpinned by a systematic review and meta-analysis of the current literature and an international consensus statement. The randomised design and assessor blinding approach will minimise selection, detection, and performance biases. A well-designed prehabilitation study will positively impact patient outcomes by improving strength and function and reduce anxiety prior to surgery thereby ensuring quicker recovery post-op. It is anticipated to reduce socio-economic burden by facilitating continuation or resumption of work and may reduce economic impact on services post-op (e.g., reduced need for physiotherapy follow-up and quicker discharge from the service). The qualitative component of this study will capture both patients and clinician perspectives of the safety, suitability and acceptability of the intervention and outcome measures and will assist in adapting the study elements prior to a full randomised control trial to test the effectiveness of prehabilitation in this population. This study is conducted within a tertiary NHS setting and therefore can be reproduced and applied in similar healthcare settings. |
| **Reviewer 3** |  |
| 19. Randomization:   More details are needed in this section, specifically, what does the authors meant by a computer-generated random allocation sequence? Furthermore, why a block randomization (often convenient and appealing in RCTs) not conducted? | This paragraphs has been edited as follows  A simple random allocation sequence will be generated using GraphPad QuickCalcs website [41] and uploaded to REDCap. Once patients are allocated to one of the two groups (prehabilitation or standard care), all baseline data will be obtained. All study personnel will be blinded to the allocation sequence ensuring adequate concealment [35, 36]. |
| 20. Sample size/Power:   The sample size/power writeup does not present the desired effect size that the investigators want to power the study upon; they only refer to a published paper. More details needed. Furthermore, it doesn't clearly state the specific statistical tests that was used to calculate the power/sample sizes. Moreover, given the apparent longitudinal nature of the study (statistical analysis will assess study outcome measures), sample sizes could have been computed, reflecting the actual study design (repetitions), keeping the same significance level, and same power. ANOVA could be used directly to compute the sample size, versus some simple 2/multiple group tests. Several software provides that option as well. Justification is missing in that regard. | A very valid point. However, we wish to point out that this study is only looking at the feasibility, acceptability and safety of the prehab programme and not testing the effectiveness of the intervention. Therefore, an appropriate power calculation has not been undertaken at this stage. The descriptive stats from this paper will be utilised to calculate the sample size for a full RCT in the future. |
| 21. Statistical Analysis:   Independent t-tests are valid only under Normality assumptions. Alternative statistical methods are not discussed, under violations to the Gaussian assumptions. Furthermore, since the study will be conducted at repeated time-points, it is not clear why a GEE, a mixed model, or even an ANOVA (repeated measures, precisely) was not proposed. Authors might be only interested in evaluating the final endpoint, however, evaluating the trajectory (since data will be available at the intermediate time-points) may strengthen the analysis. | Only descriptive statistics to evaluate the distribution of scores and calculating the floor and ceiling effects of the primary outcome measure will be analysed at this stage. |
| 22. Writing style:   Discussion Section: Given the study was designed only for UK subjects, the Discussion section should clearly allude to future studies with other populations and geographical regions to further validate their current findings. Currently, the findings are only limited to this population. | Agree. A sentence has been added to reflect this under limitation of the study. This is as follows:  The study is conducted in a single tertiary hospital in the UK and therefore may need to be adapted prior to wider implementation in other healthcare services across the globe. |
